# Regional environmental differences significantly affect the genetic structure and genetic differentiation of *Carpinus tientaiensis* Cheng, an endemic and extremely endangered species from China

**DOI:** 10.3389/fpls.2024.1277173

**Published:** 2024-02-09

**Authors:** Runan Zhao, Qianqian He, Xiaojie Chu, Anguo He, Yuanlan Zhang, Zunling Zhu

**Affiliations:** ^1^ College of Landscape Architecture, Nanjing Forestry University, Nanjing, China; ^2^ Co-Innovation Center for Sustainable Forestry in Southern China, Nanjing Forestry University, Nanjing, China; ^3^ Research Center for Urban and Rural Living Environment, Zhijiang College of Zhejiang University of Technology, Shaoxing, China; ^4^ College of Life Sciences, Zhejiang Normal University, Jinhua, China; ^5^ Administration of Zhejiang Dapanshan National Nature Reserve, Pan’an, China; ^6^ College of Life Sciences, Nanjing Forestry University, Nanjing, China; ^7^ Jinpu Research Institute, Nanjing Forestry University, Nanjing, China

**Keywords:** *Carpinus tientaiensis*, endangered species, phylogeography, genetic variation, genetic differentiation, molecular marker, ecological niche model, population dynamics

## Abstract

Differences in topography and environment greatly affect the genetic structure and genetic differentiation of species, and endemic or endangered species with limited geographic ranges seem to be more sensitive to changes in climate and other environmental factors. The complex topography of eastern China is likely to affect genetic differentiation of plants there. *Carpinus tientaiensis* Cheng is a native and endangered plants from China, and exploring its genetic diversity has profound significance for protection and the collection of germplasm resources. Based on AFLP markers, this study found that *C. tientaiensis* has low genetic diversity, which mainly came from within populations, while Shangshantou and Tiantai Mountain populations have relatively high genetic diversity. The Nei genetic distance was closely related to geographical distance, and temperature and precipitation notablely affected the genetic variation and genetic differentiation of *C. tientaiensis*. Based on cpDNA, this study indicated that *C. tientaiensis* exhibits a moderate level of genetic diversity, and which mainly came from among populations, while Tiantai Mountain population have the highest genetic diversity. It demonstrated that there was genetic differentiation between populations, which can be divided into two independent geographical groups, but there was no significant phylogeographic structure between them. The MaxEnt model showed that climate change significantly affects its distribution, and the suitable distribution areas in Zhejiang were primarily divided into two regions, eastern Zhejiang and southern Zhejiang, and there was niche differentiation in its suitable distribution areas. Therefore, this study speculated that the climate and the terrain of mountains and hills in East China jointly shape the genetic structure of *C. tientaiensis*, which gived rise to an obvious north-south differentiation trend of these species, and the populations located in the hilly areas of eastern Zhejiang and the mountainous areas of southern Zhejiang formed two genetic branches respectively.

## 1 Introduction

Topographic and environmental differences, which also promote the formation of genetic structures, shape the genetic variation between or within species (Hewitt, 2000; Hewitt, 2004; Zhang et al., 2020). Researches have indicated that intraspecific population differentiation and genetic structure are significantly influenced by geological and climate differences (Zhang et al., 2013a; Zhang et al., 2013b; Sun et al., 2014; Kou et al., 2016), and endemic species are particularly vulnerable to specialized ecological niches because of limited distribution range, so they are extremely sensitive to climate change, geological vicissitude, and human destruction (Robin et al., 2010; Chen et al., 2020; Hou et al., 2020; Sękiewicz et al., 2020). Additionally, genetic differentiation and structure of species may also be affected by isolation by distance and environment (Meng et al., 2017; Xing and Ree, 2017; Guo et al., 2023). There are a variety of complex landscapes such as mountains, hills, and plains in East China. During the Quaternary glacial period, only a few mountains in this region developed glaciers (Zhou et al., 2011). The unique geological and environmental circumstances there provide unique advantages for the evolution of woody plants (Lu et al., 2018). The adjacent areas of Zhejiang and Anhui, as well as the regions of south-west Zhejiang, are also considered as centres of plant endemism in China (López-Pujol et al., 2011).

Genetic diversity of species is the product of evolution and long-term adaptation to its surrounding environment (De Kort et al., 2021; Lande, 1988), and it is also a necessary prerequisite for survival and development of species. Genetic diversity can reflect the richness of genetic variation of species. When species have high genetic diversity, their adaptability to the environment is stronger, while on the contrary, it is weaker (Lande, 1988; Frankham, 2005; DeWoody et al., 2021). It is the core component of biodiversity, and the protection of biodiversity is ultimately the protection of genetic diversity, so genetic diversity has become one of the main research contents of conservation biology (Frankham, 2005; Laikre et al., 2020; Hoban et al., 2022). The research of genetic variation and structure of species can provide a scientific foundation and theoretical guidance for species protection and utilization. The research on genetic diversity of endangered or rare species can understand not only the origin and evolution mechanism of these species, but also the ecological adaptation mechanism at the molecular level, and provide theoretical basis for species protection and genetic diversity conservation (Holsinger and Gottlieb, 1991; Wang et al., 2020; Ma et al., 2021).

Chloroplast DNA (cpDNA) can be inherited independently (Neuhaus and Emes, 2000; Camus et al., 2022; Laurentin Táriba, 2023), does not participate in gene recombination, and is not interfered by gene overlap and deletion. It has an independent evolutionary route, a small molecular weight, and a straightforward structure (Kusnetsov, 2018; Dobrogojski et al., 2020). It is frequently used to reconstruct the relationship between species, intra-species phylogeographic differentiation, species distribution pattern and historical dynamics. It is one of the most efficient and commonly utilized markers for studying genetic variation and lineage divergence of plant (Petit et al., 1997; Mahadani et al., 2022; Antil et al., 2023). Amplified fragment length polymorphism (AFLP) marker offers the advantages of great dependability, stable experimental results, no need to know the DNA sequence beforehand, and only a few primers can cover the entire genome (Vos et al., 1995). AFLP markers can be used to examine genetic structure, genetic differentiation, and geographical distribution pattern of genetic diversity of species (Wu et al., 2020; Adhikari et al., 2022; Xu et al., 2022). It is of utmost practical relevance for the protection and utilization of endangered species to clarify the internal and external factors that affect endangerment or even extinction and establish corresponding conservation strategies based on genetic variation and structure.

*Carpinus tientaiensis* Cheng is an endemic endangered species from the Tertiary period in China and only distributes in the mountainous and hilly areas of eastern and southern Zhejiang (Cheng, 1932; Li and Zheng, 1979; Li and Skvortsov, 1999). It has a very small number of natural individuals, and has been listed on the IUCN (International Union for Conservation of Nature) Red List of Threatened Species with an endangered status of CR (critically endangered) (Shaw et al., 2014). It has been discovered that there is genetic differentiation between populations of *C. tientaiensis* based on cpDNA (Chen and Yang, 2022), but the characteristics of genetic differentiation and spatial distribution pattern of genetic variation among populations are not clear, and its genetic diversity, genetic structure, and population history dynamics still need to be elucidated. At the nuclear gene level, is there genetic differentiation between populations of *C. tientaiensis*? What is the level of genetic diversity in its population, and are there significant differences in genetic diversity among populations? Based on the MaxEnt model, it was found that *C. tientaiensis* is highly sensitive to climate change, and its suitable distribution and core suitable areas will decrease sharply in future climate change scenarios (Zhao et al., 2020). What was the distribution of *C. tientaiensis* in the past historical periods? And whether paleo climate change has affected its distribution? These issues have not yet been clarified.

In addition, comprehension of the influence of geographical and environmental differences on genetic structure and differentiation is also helpful to protect endangered species. Therefore, this study aims to: 1) analyze its genetic diversity and verify whether there is population differentiation; 2) analyze genetic differentiation and structure among populations; 3) explore whether the environmental characteristics of eastern China affect its genetic diversity and structure; 4) analyze its population dynamics history and explore whether climate change has affected its population distribution.

## 2 Materials and methods

### 2.1 Collection of materials

A thorough investigation was conducted in Zhejiang and its adjacent areas between 2018 and 2020. Finally, a total of 53 individual were collected from Tiantai Mountain (TTS), Dapan Mountain (DPS), Yangtianhe (YTH), and Shangshantou (SST) (**Supplementary Table S1**). The collected molecular materials were immediately stored in preservation bags containing silica gel. The voucher specimens for each population were kept in the landscape experimental training center at Nanjing Forestry University, Nanjing, China. The DNA of *C. tientaiensis* was extracted using the modified CTAB method (modified from Doyle and Doyle, 1987).

### 2.2 Amplification and analysis of AFLP markers

#### 2.2.1 Experimental process

The experiment process was adjusted based on Vos et al. (1995). The digestion reaction system was 20 μL (**Supplementary Table S2**), with *EcoR* I and *Mse* I (**Supplementary Table S3**) as restriction endonucleases, and 10×Buffer was used as buffer for digestion and connection. After mixing the above reaction liquid, centrifuge it at 12,000 rpm for 30 s, then hold it at 37 °C for 5 hours, at 8 °C for 4 hours, and finally store it overnight at 4 °C. Subsequently, the double enzyme digestion products of each sample were examined by electrophoresis, and the findings of enzyme digestion electrophoresis were examined to observe whether DNA bands were dispersed and whether the digestion was complete.

The pre-amplification reaction system was 20 μL (**Supplementary Tables S4, S5**). After centrifugation of the digestion and connection product, the product was pre-denatured at 94 °C for 2 min. Subsequently, they were amplified at 94 °C for 30 s, 56 °C for 30 s, and 72 °C for 80 s. The amplification cycle was 30 rounds, and then extended at 72 °C for 5 min. Diluted the pre-amplified product with AFLP-TE at 1:20 and used them as the template for selective PCR amplification (**Supplementary Tables S6, S7**).

After gel examination, 8 pairs of primer combinations (**Supplementary Table S8**) with a large number of bands, high polymorphism, and easy interpretation were selected for further amplification. The reaction procedure for the subsequent first round of amplification was to pre-denature at 94 °C for 2 min, then amplify at 94 °C for 30 s, amplify at 65 °C for 30 s, and then amplify 72 °C for 80 s. The first round of amplification consisted of 14 cycles, and the temperature of each round of cyclic annealing decreased by 0.7 °C. Afterwards the amplification was carried out at 94 °C for 30 s, 55 °C for 30 seconds, and 72 °C for 80 s. After 23 rounds of amplification, it was extended at 72 °C for 5 min. After the reaction, the amplified product was mixed with formamide loading solution at 3:8, and then denatured at 95°C for 5 min, followed by immediate ice bath. After 30 min of pre-electrophoresis, 7 μL of samples were taken for electrophoresis detection.

#### 2.2.2 Data analysis

The electrophoresis data was subsequently extracted by GENESCAN v3.1 software (Applied Biosystems, CA, USA). The size of each segment was extracted by the Binthere software (Applied Biosystems, CA, USA), which was then translated to the matrix data made up of “0” and “1”. To measure the amplified site polymorphism, POPGENE v1.32 software (http://www.ualberta.ca/~fyeh/) was used to calculate the number of polymorphic loci (*N*) and percentage of polymorphic loci (*PPBs*) for the total population and each pair of primers and population. The observed number of alleles (*Na*), effective number of alleles (*Ne*), Nei’s gene diversity (*H*), Shannon’s Information index (*I*), total genetic diversity (*Ht*), and within population genetic diversity (*Hs*) were calculated to evaluate the degree and sources of genetic variation of each pair of primers and population. And the genetic differentiation (*Gst*) and gene flow (*Nm*) of each pair of primers were used to judge whether there was population differentiation and phylogeographic structure.

The spatial interpolation analysis of population genetic diversity was conducted by ArcGIS v10.2 (ESRI Inc., Redlands, CA, USA), based on *H* and inverse distance weighting (IDW) method. Bayesian cluster analysis and admixture model were conducted by Structure v2.3.4 software (Pritchard et al., 2000) to evaluate genetic structure of *C. tientaiensis*. The genetic grouping (*K*) was set to 1-10 and the number of iterations was 10. The optimal number of groups of *C. tientaiensis* populations was determined by the Delta *K* method in the online tool of Structure Harvester (Earl and vonHoldt, 2012). The results of 10 runs were then reprocessed by CLUMPP v1.1.2 (Jakobsson and Rosenberg, 2007) to calculate the provenance coefficient (*q*) of each population and individual assigned to various genetic groups. The genetic structure map was created with Distuct v1.1 software (Rosenberg, 2004), and the distribution map was made with ArcGIS v10.2.

Analysis of molecular variance (AMOVA) was performed using Arlequin v3.5 (Excoffier and Lischer, 2010) to investigate the sources of genetic variation. The Similarity program in NTSYSpc v2.11F software (Rohlf, 2000) was used to calculate Nei genetic distance among each individual, and the Mantel test was performed using GeneALEx v6.51b2 software (Smouse et al., 2017) to evaluate whether Nei genetic distance was related to geographic distance (GGD). Subsequently, 19 bioclimatic variables were achieved from Worldclim (Fick and Hijmans, 2017) and pearson correlation analysis was conducted on the related genetic diversity index and bioclimatic and geographical variables using Origin v2022b (Electronic Arts Inc., CA, USA).

### 2.3 Amplification, sequencing and analysis of cpDNA

#### 2.3.1 Amplification and sequencing

In this research, eight cpDNA sequences (**Supplementary Table S9**) were selected for PCR amplification and sequencing. The amplification system was 30 μL, including 1 μL DNA of *C. tientaiensis*, 27 μL Tsingke PCR Mix, 1 μL forward and reverse primers, and 1 μL template DNA. The products were examined by electrophoresis, and then the products were two-way sequenced by Tsingke Biotechnology Co., Ltd. (Beijing, China). Among the 8 sequences, 5 sequences failed to amplify or failed to discover obvious mutation sites. Therefore, the primers of *trn*L-*trn*F (Sang et al., 1997), *trn*G (Nishizawa and Watano, 2000) and *psb*A-*trn*H (Okaura et al., 2007) were selected for amplification and sequencing of all individuals, and finally the sequences of 52 individual were obtained.

#### 2.3.2 Data analysis

The sequencing results were manually corrected using SeqMan Pro v11.1.0 software (DNASTAR Inc., Madison, USA), and then all sequences were aligned by MEGA v10.1.7 software (Kumar et al., 2018), and finally three sequences were spliced into a single sequence. DnaSP v5.10 (Librado and Rozas, 2009) was used to determine the number of chloroplast haplotype, and calculate haplotype diversity (*H*_d_), nucleotide polymorphism (π), and genetic differentiation between populations (*GammaSt*). The haplotypes sequences have been uploaded to GenBank (https://www.ncbi.nlm.nih.gov/genbank/, accession numbers: OR934485 to OR934497). Subsequently, Mantel test was performed using GeneALEx v6.51b2 software (Smouse et al., 2017) to evaluate the correlation between *GammaSt* and GGD, and gene flow (*Nm*) was estimated according to Chen et al. (2008). The median-joining (MJ) network of chloroplast haplotypes was created and relationship between chloroplast haplotypes was analyzed by POPART v1.7 software (Leigh and Bryant, 2015). Then, phylogenetic relationship based on the Neighbor-Joining (NJ) method was analyzed using MEGA v10.1.7 (Kumar et al., 2018), with 1000 bootstrap replicates.

Similarly, Arlequin v3.5 software (Excoffier and Lischer, 2010) was used to conduct AMOVA to examine the sources of genetic variation. PERMUT 2.0 software (Pons and Petit, 1996) was used to calculate genetic diversity (*h*_T_), average genetic diversity within populations (*h*_S_), and genetic differentiation (*G*_ST_ and *N*_ST_), and then compare the differences between *G*_ST_ and *N*_ST_ using 10000 times of replacement tests in order to ascertain whether the populations have significant phylogeographic structure. The spatial interpolation analysis of population genetic diversity based on *H*_d_ and IDW method (Bartier and Keller, 1996) was performed using ArcGIS v10.2. To determine if the population has experienced expansion, mismatch distribution analysis (MDA), Fu’s *Fs* test, and Tajima’s *D* test were analyzed by DNASP v5.10 (Librado and Rozas, 2009).

### 2.4 Geographical distribution and historical dynamics

The MaxEnt v3.4.4 software (Phillips et al., 2023) was used to reconstruct the current and paleo ecological niches of *C. tientaiensis*. The climate data for the Last Interglacial (LIG, about 120,000-140,000 years BP), Mid-Holocene (Mid, about 6,000 years BP), and the current (1,970s-2,000s) were obtained from the Worldclim (Fick and Hijmans, 2017). The climate data for the Last Glacial Maximum (LGM, about 21,000 years BP) were obtained from the CHELSA (Climatologies at high resolution for the earth’s land surface areas) (Karger et al., 2021). The climate data for these four periods all included 19 bioclimatic variables (**Supplementary Table S10**) with a spatial resolution of 30”. Due to the differences among different Global Climate Models (GCMs), three GCMs including CCSM4 (Community Climate System Model version 4, CC), MIROC-ESM (the Model for Interdisciplinary Research on Climate, Earth System Model, MR) and MPI-ESM-P (the Max Planck Institute for Meteorology Earth System Model, ME) were selected for Mid and LGM periods.

In the field investigation, the 2bulu software (https://www.2bulu.com/) was used to record the location information of each individual. In order to avoid overfitting caused by clustering of distribution points as much as possible, only one distribution point was selected in every 30”×30” region, and finally obtained a total of 12 effective distribution data (**Supplementary Table S11**). Due to the narrow distribution range of *C. tientaiensis*, and the geographical range of the bioclimatic variables can affect the accuracy of the model, the bioclimatic variables were limited to the geographical range of approximately 21°N-33°N and 105°E-123°E. In order to minimize errors caused by the correlation between bioclimatic variables, the spatial analysis tools in ArcGIS v10.2 software were used to analyze the correlation between the bioclimatic variables (**Supplementary Table S12**). When the correlation coefficient was ≥ |0.85|, the bioclimatic variables with higher ecological significance for constructing model were retained, based on the jackknife analysis results of MaxEnt model (**Supplementary Table S10**). Finally, 9 bioclimatic variables were selected for the construction of the model (**Supplementary Table S13**).

The MaxEnt model (Phillips et al., 2017) was widely used to reconstruct potential habitats of species, and was still effective even when there were less than 10 occurrence records. However, when the sample sizes of the occurrence records were extremely small, using only default parameters may lead to errors in the prediction results. Therefore, the delete-one jackknife approach (Pearson et al., 2007, Peterson et al., 2011, Kass et al., 2022) was applied to establish model, and the regularization multiplier was adjusted to weaken the bias caused by few occurrence records. The models were built used linear+quadratic features under different regularization multipliers (0.25, 0.3, 0.4, 0.5, 0.6, 0.7, 0.75, 1, 1.25, 1.5, 1.75, 2), and the area under the curve (AUC), minimum training presence area, maximum test sensitivity plus specificity area, and equate entropy of thresholded and original distributions area were used to determine the optimal model (**Supplementary Table S14**). Finally, the regularization multiplier was determined to be 0.5.

The logistic thresholds of “equate entropy of thresholded and original distributions” and “maximum training sensitivity plus specificity” were used as the thresholds of suitable distribution areas (SDAs) and core suitable areas (CSAs), respectively. Based on ArcGIS v10.2, the public areas in which all the three GCMs in Mid and LGM periods were SDAs and CSAs were extracted respectively. At the same time, the average values of the SDAs and CSAs for the three GCMs in Mid and LGM periods were calculated, respectively. The public areas of the SDAs in the four periods were extracted using ArcGIS v10.2, and the mean center of the SDAs in the four periods were calculated in order to express the change trend of the SDAs.

## 3 Results

### 3.1 Genetic diversity and genetic differentiation based on AFLP markers

#### 3.1.1 Amplified site polymorphism

A total of 1728 loci were amplified, of which 1316 were *N*, and *PPBs* was about 76.16% (**Supplementary Table S15**). The average *N* amplified by each pair of primers was 165, of which the efficiency of *E-ACG/M-CAT* primer combination was the lowest, the *N* amplified was 153, and *PPBs* was about 70.83%. The efficiency of *E-AGC/M-CTC* primer combination was the highest, the *N* amplified was 179, and *PPBs* was about 82.87% (**Supplementary Table S15**). Among the populations, the *N* amplified by SST population was the largest, with an average of 137 loci, and *PPBs* was about 63.48%. The *N* amplified by YTH population was the least, with an average of 35 loci, and *PPBs* was about 16.09% (**Supplementary Table S15**).

#### 3.1.2 Genetic diversity

The average *Na*, *Ne*, *H* and *I* of *C. tientaiensis* were about 1.7616, 1.2331, 0.1501 and 0.2453, respectively. The highest *Ne* (1.2181) and *H* (0.1377) were observed in TTS population, while the highest *Na* (1.6348) and *I* (0.2220) were observed in SST population. All genetic diversity indexes of YTH population were the lowest (*Na*=1.1609、*Ne*=1.1138、*H*=0.0666、*I=*0.0973). Therefore, TTS and SST populations had significant genetic diversity, while YTH population had low genetic diversity (**Supplementary Table S16**).

#### 3.1.3 Genetic differentiation

The *Ht* of *C. tientaiensis* was about 0.1516, and *Hs* was about 0.1165 (**Supplementary Table S17**). The results of AMOVA revealed that genetic variation among populations was approximately 15.47% (**Supplementary Table S18**). It can be found that *C. tientaiensis* has a low total genetic diversity, with which mainly came from within populations. The genetic differentiation (*Gst*), which was about 0.2333, indicating there was genetic differentiation between populations. There was a significant amount of gene exchange between populations, as seen by the gene flow (*N*_m_), which is approximately 1.6576 (**Supplementary Table S17**). Therefore, there was no remarkable phylogeographic structure between populations of *C. tientaiensis*.

**Figure 1:**
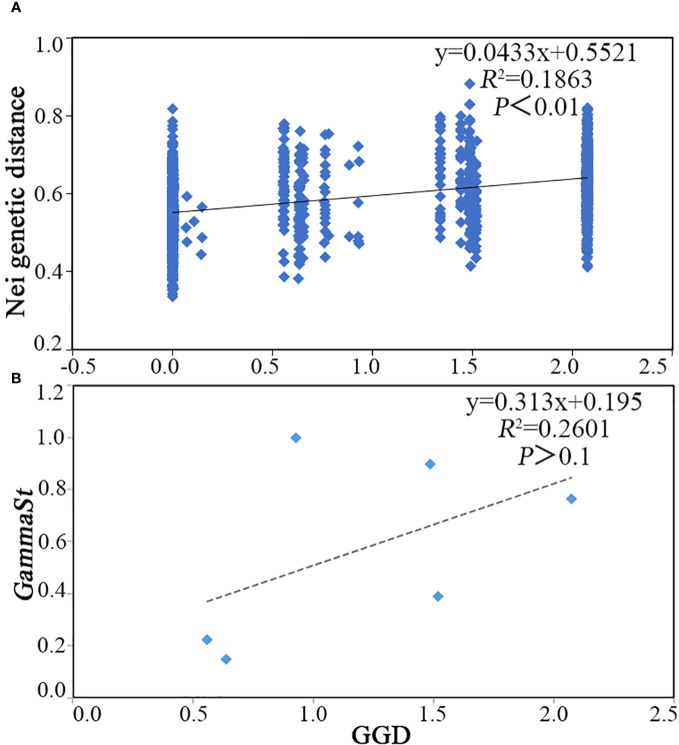
Mantel test of *C. tientaiensis*. **(A)** Nei genetic distance and geographic distance (GGD) based on AFLP Markers; **(B)** *GammaSt* and GGD based on cpDNA.

**Figure 2:**
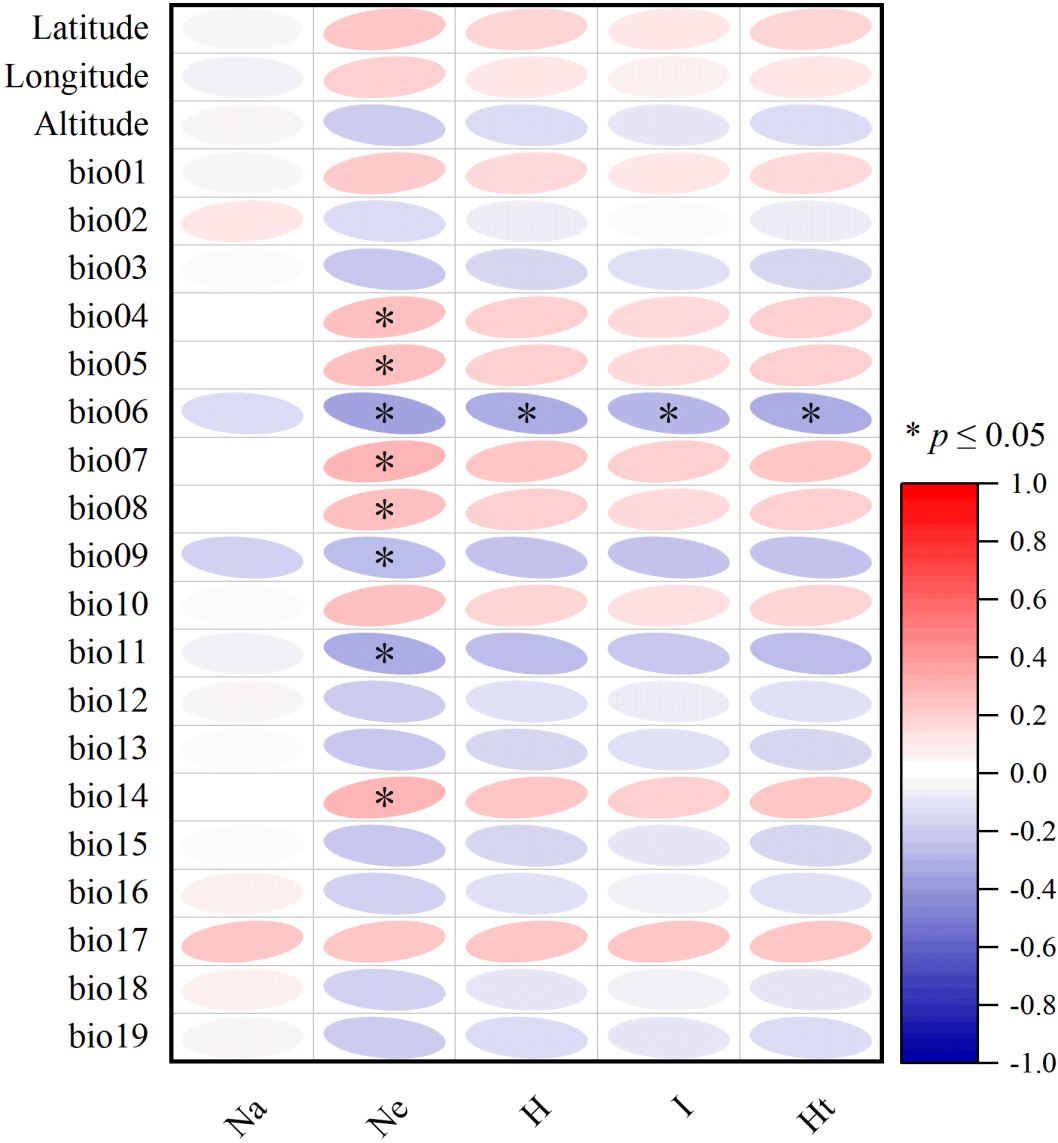
Correlation heat map between genetic diversity index and geographical and bioclimatic variables. * indicate significant correlation at the p ≤ 0.05 level.

As geographical distance increased, the Nei genetic distance increased as well (*R* ^2 =^ 0.1863, *P*<0.01) (**Figure 1A**). Subsequently, the correlation between genetic diversity index and bioclimatic and geographical variables was tested, and it was found that genetic diversity index had little correlation between latitude, longitude, and altitude (**Figure 2**). Nonetheless, *Ne* was significantly negatively correlated with bio06, bio09, and bio11, as well as a significant positive correlation with bio04, bio05, bio07, bio08, and bio14 (**Figure 2**). In addition, *H*, *I*, and *Ht* were all significantly negatively correlated with bio06 (**Figure 2**).

#### 3.1.4 Population genetic structure

**Figure 3:**
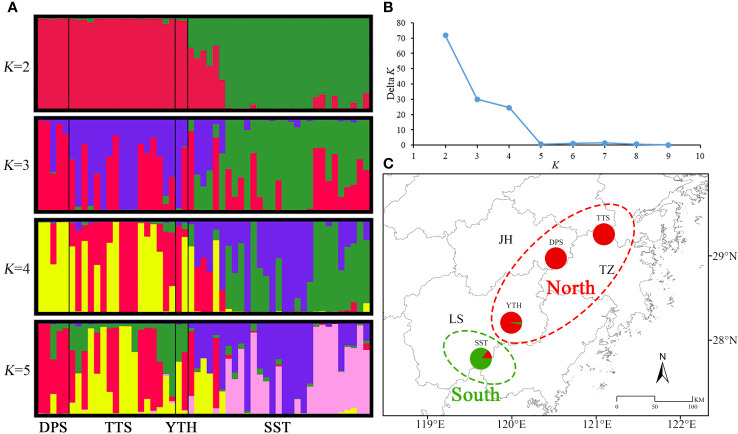
Bayesian clustering results of *C. tientaiensis.* **(A)** Composition of genetic branches for each individual when *K*=2, 3, 4, and 5; **(B)** Delta *K*; **(C)** Bayesian clustering results when *K*=2. JH, Jinhua city; LS, Lishui city; TZ, Taizhou city; TTS, Tiantai Mountain, Taizhou city; DPS, Dapan Mountain, Jinhua city; YTH, Yangtianhe, Lishui city; SST, Shangshantou, Lishui city.

The Bayesian clustering results indicated that the optimal grouping number of *C. tientaiensis* was 2 (**Figure 3B**), and at this moment, the populations can be separated into two independent geographical groups (**Figures 3A, C**). Among which, the northern geographical group included TTS, DPS and YTH populations. The provenance coefficient (*q*_w_) of this group from the northern genetic branch was greater than 0.9. The southern geographical group only included SST population, and the *q*_w_ from the northern genetic branch was less than 0.7. The genetic backgrounds of TTS population was displayed when *K*=3. When *K*=4, both TTS and SST populations primarily had two genetic backgrounds. When *K*=5, TTS and SST populations mostly had three and two genetic backgrounds respectively.

### 3.2 Genetic structure and historical dynamics based on cpDNA

#### 3.2.1 Genetic diversity

After sequence alignment, the length of *trn*L- *trn*F, *trn*G and *psb*A-*trn*H were 452 bp, 603 bp and 514 bp respectively, and the length of spliced sequence was 1569 bp. A total of 9 chloroplast haplotypes and 10 polymorphic sites were identified in the sequence, including 2 singleton variable sites and 8 parsimony informational sites (**Supplementary Table S19**). The total *H*_d_ of *C. tientaiensis* was 0.6825 ± 0.063, and the total π was 1.81 ± 0.14×10^-3^. The *H*_d_ of each population ranged from 0 to 0.757 ± 0.063, and π ranged from 0 to 1.03 ± 0.14×10^-3^. Among which, TTS population had the highest *H*_d_ (0.757 ± 0.063) and π (1.03 ± 0.14×10^-3^), while DPS and YTH populations had the lowest *H*_d_ (0) and π (0) (**Supplementary Table S20**).

#### 3.2.2 MJ network of haplotypes and phylogenetic relationship

**Figure 4:**
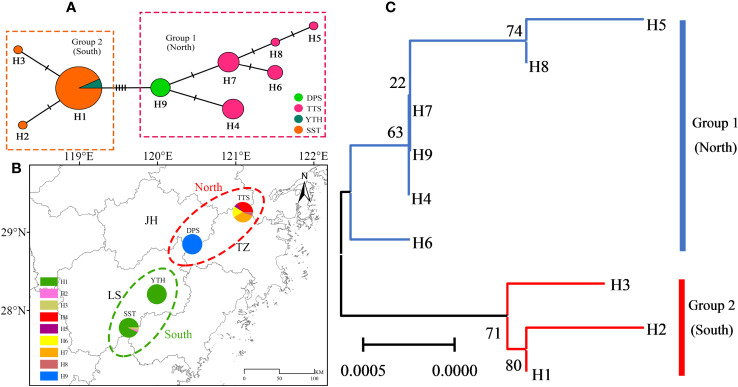
The median-joining (MJ) network and phylogenetic relationship of chloroplast haplotypes. **(A)** MJ network; **(B)** geographical distribution; **(C)** phylogenetic relationships. JH, Jinhua city; LS, Lishui city; TZ, Taizhou city; TTS, Tiantai Mountain, Taizhou city; DPS, Dapan Mountain, Jinhua city; YTH, Yangtianhe, Lishui city; SST, Shangshantou, Lishui city.

Among the nine identified chloroplast haplotypes (H1-H9), the frequency of H1 was the highest (28/52 = 53.85%), followed by H4 (6/52 = 11.54%) and H7 (6/52 = 11.54%), with H2, H3, H5, and H8 being unique haplotypes (**Supplementary Tables S19**, **S20**). According to the MJ network and phylogenetic relationship of chloroplast haplotypes (**Figure 4**), the populations of *C. tientaiensis* can be separated into two separate geographical groups. Among which, the northern geographical group included TTS and DPS populations. This group contained six chloroplast haplotypes, with H4 (6/22 = 27.27%) and H7 (6/22 = 27.27%) serving as the most prevalent. Moreover, H9 was the unique haplotype of DPS population. The southern geographical group consisted of YTH and SST populations. There were only three chloroplast haplotypes in this group, and H1 accounted for 93.33% of all individuals (**Figure 4**; **Supplementary Table S20**).

#### 3.2.3 Gene flow and spatial pattern of molecular variation

**Figure 5:**
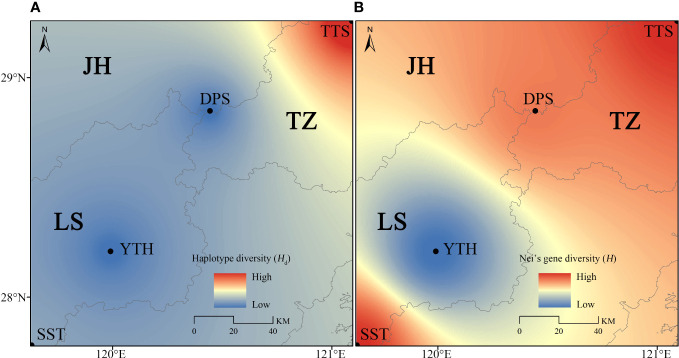
Spatial interpolation analysis of *C. tientaiensis*. **(A)** Based on haplotype diversity (*H*_d_); **(B)** Based on Nei’s gene diversity (*H*). JH, Jinhua city; LS, Lishui city; TZ, Taizhou city; TTS, Tiantai Mountain, Taizhou city; DPS, Dapan Mountain, Jinhua city; YTH, Yangtianhe, Lishui city; SST, Shangshantou, Lishui city.

The *N*_m_ between populations ranged from 0 to 2.083. Among which, TTS and DPS populations had the most active gene flow (*N*_m_), which suggested that their genetic differentiation was minimal (**Supplementary Figure S1**). Moreover, there was active gene flow (*N*_m_) between YTH and SST populations. As a result, the northern geographical group had more vigorous gene flow (**Supplementary Figure S1**). The genetic diversity of populations exhibited a north-south differentiation tendency, according to spatial interpolation analysis (**Figure 5**). The northern geographical group had relatively high genetic diversity, while the southern geographical group had relatively low genetic diversity (**Figure 5**).

#### 3.2.4 Genetic differentiation

The results of AMOVA revealed that there was obvious genetic differentiation in the populations of *C. tientaiensis*, with the genetic variation among populations being around 85.55% and the genetic variation within populations being approximately 14.44% (**Supplementary Table S18**). The total *H*_T_ of *C. tientaiensis* was 0.852, and *H*_S_ was 0.243. The genetic differentiation coefficient *G*_ST_ and *N*_ST_ of *C. tientaiensis* were 0.714 and 0.857, respectively (*p*>0.05). The *N*_ST_ was somewhat greater than *G*_ST_, but neither *N*_ST_ nor *G*_ST_ were statistically significant, demonstrating that there was no significant phylogeographic structure. As geographical distance increased, the *GammaSt* increased as well (*R* ^2 =^ 0.2601, *P*>0.1) (**Figure 1B**).

#### 3.2.5 Population historical dynamics

**Figure 6:**
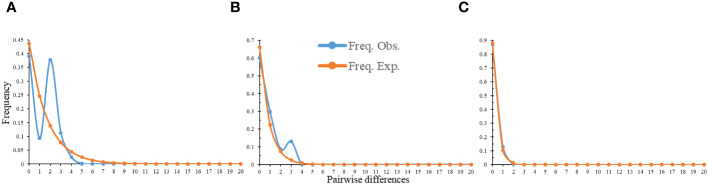
The results of mismatch distribution analysis (MDA) of *C. tientaiensis.* **(A)** the total population; **(B)** northern geographical group; **(C)** southern geographical group.

There were two peaks on the distribution mismatch curve of the total population and northern geographical group, but only one peak in the southern geographical group (**Figure 6**). Furthermore, the neutral test results of the total population, northern geographical group and southern geographical group were all negative value and not statistically significant (**Supplementary Table S21**). Therefore, it appeared that there may not be a large-scale expansion of *C. tientaiensis*.

### 3.3 Historical changes of geographical distribution

**Figure 7:**
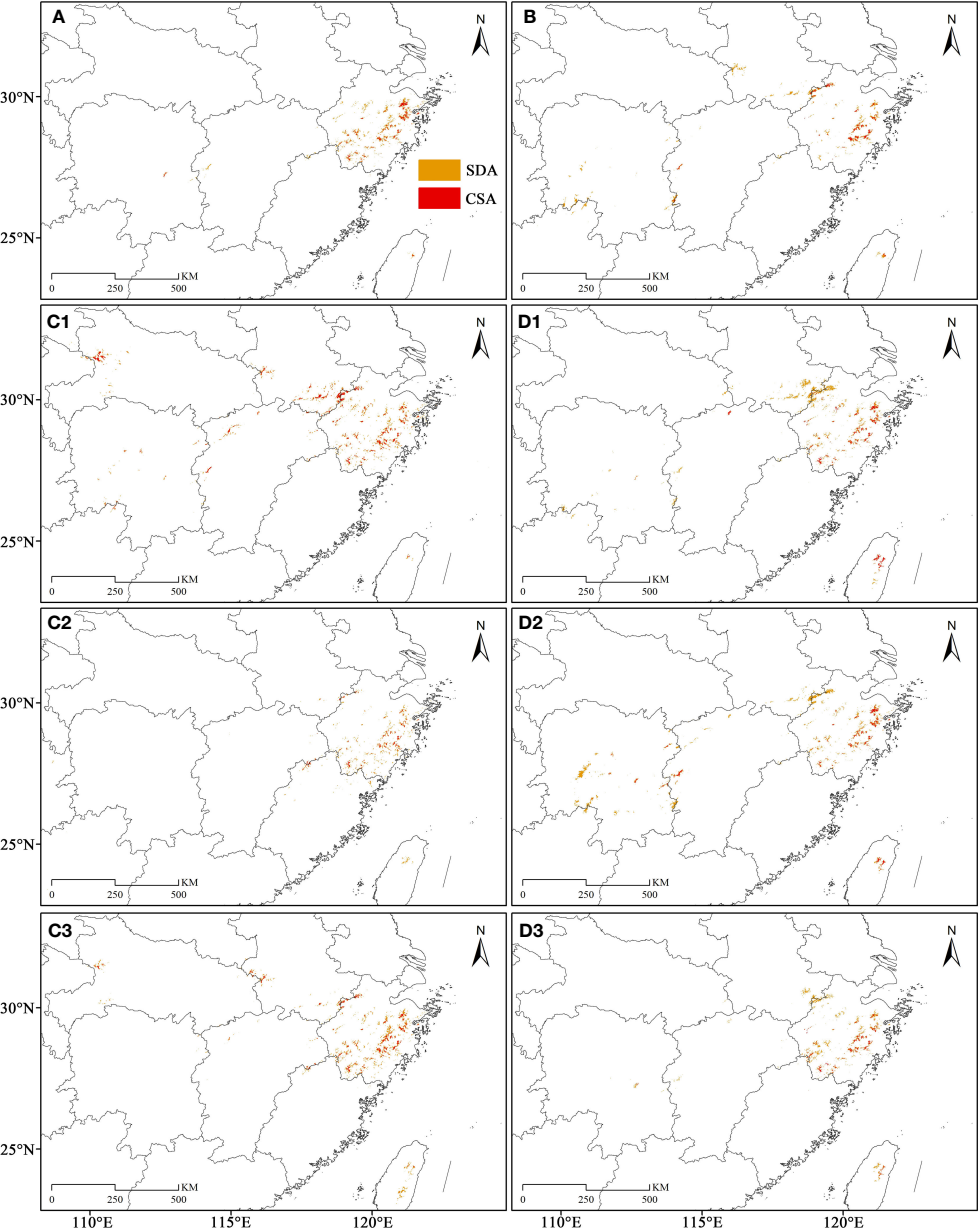
The paleo and current potential geographical distribution of *C. tientaiensis*. **(A, B)** The SDAs (suitable distribution areas) and CSAs (core suitable areas) during the LIG (the Last Interglacial, about 120,000-140,000 years BP) and current periods, respectively. **(C1-3, D1-3)** The SDAs and CSAs under three GCMs (Global Climate Models) of CC (Community Climate System Model version 4, CCSM4), MR (the Model for Interdisciplinary Research on Climate, Earth System Model, MIROC-ESM) and ME (the Max Planck Institute for Meteorology Earth System Model, MPI-ESM-P) during the LGM (Last Glacial Maximum, about 21,000 years BP) and Mid (Mid-Holocene, about 6,000 years BP) periods, respectively. SDA, suitable distribution area; CSA, core suitable area.

**Figure 8:**
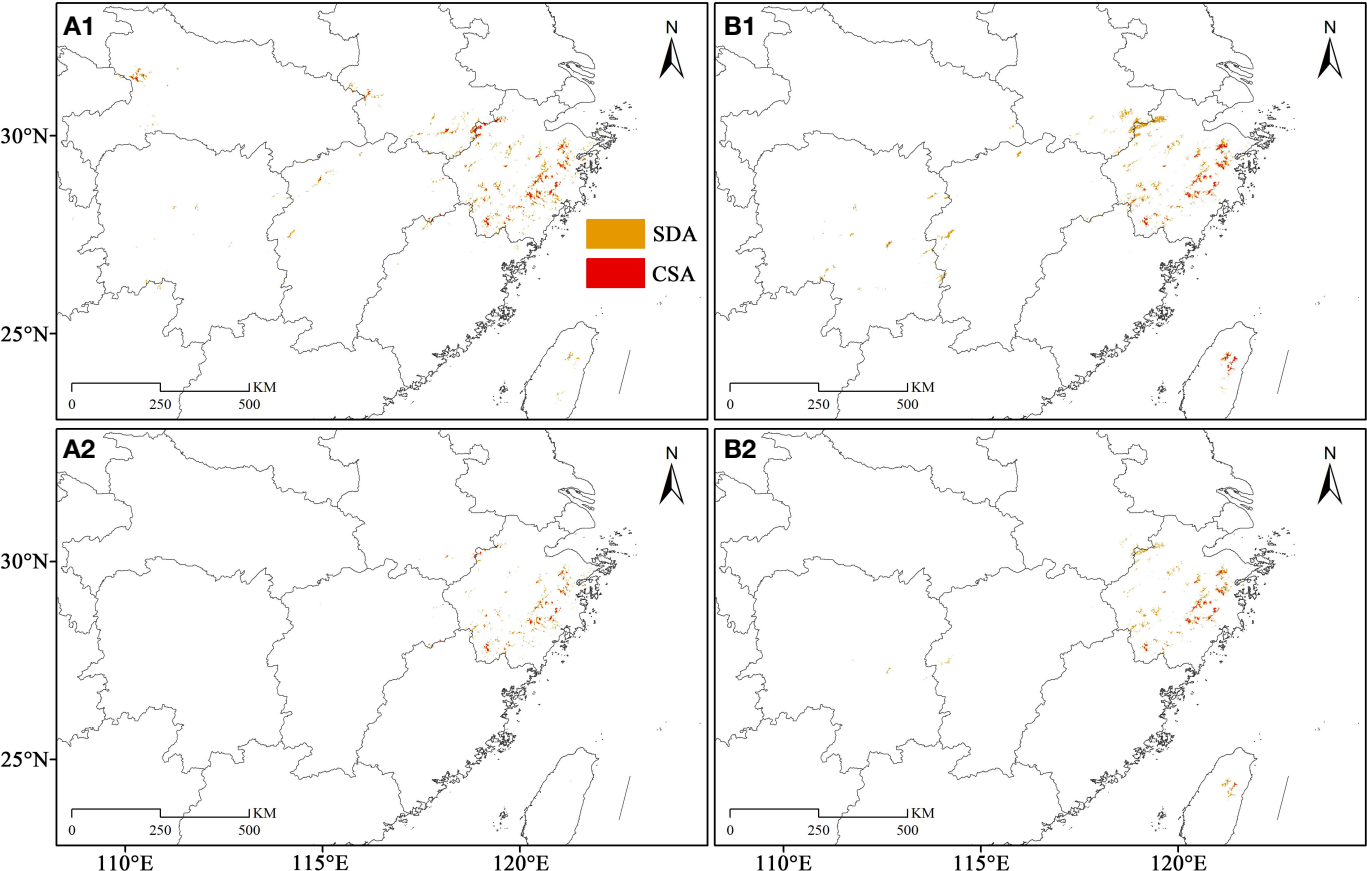
The potential geographical distribution of *C. tientaiensis* in the LGM (Last Glacial Maximum, about 21,000 years BP) and Mid (Mid-Holocene, about 6,000 years BP) periods. **(A1, B1)** The average values of SDAs (suitable distribution areas) and CSAs (core suitable areas) in three GCMs (Global Climate Models) during LIG and Mid, respectively. **(A2, B2)** The regions that were all SDAs and CSAs in three GCMs during the LIG and Mid periods, respectively. SDA, suitable distribution area; CSA, core suitable area.

The SDAs and CSAs were primarily concentrated in northeastern Zhejiang, while in eastern and southwestern Zhejiang presented a merely scattered distribution trend, during the LIG (**Figure 7A**). During the LGM, its SDAs and CSAs in northeastern Zhejiang decreased significantly, but there were some SDAs and CSAs in the northwest of Zhejiang (**Figures 7C1-C3**, **8A1-A2**, **9A**). The SDAs and CSAs in Zhejiang were mainly scattered in eastern and southern Zhejiang (**Figures 7C1-C3**; **8A1-A2**, **9A**). Throughout the Mid, the SDAs and CSAs were primarily found in northeast and east of Zhejiang (**Figures 7D1-D3**, **8B1-B2**, **9B**), with an increasing trend of SDAs in eastern Zhejiang (**Figures 8B2**, **9B**). In current climate scenario, the SDAs and CSAs in Zhejiang were primarily concentrated in the east and northeast, while in the south were significantly reduced (**Figure 7B**).

**Figure 9:**
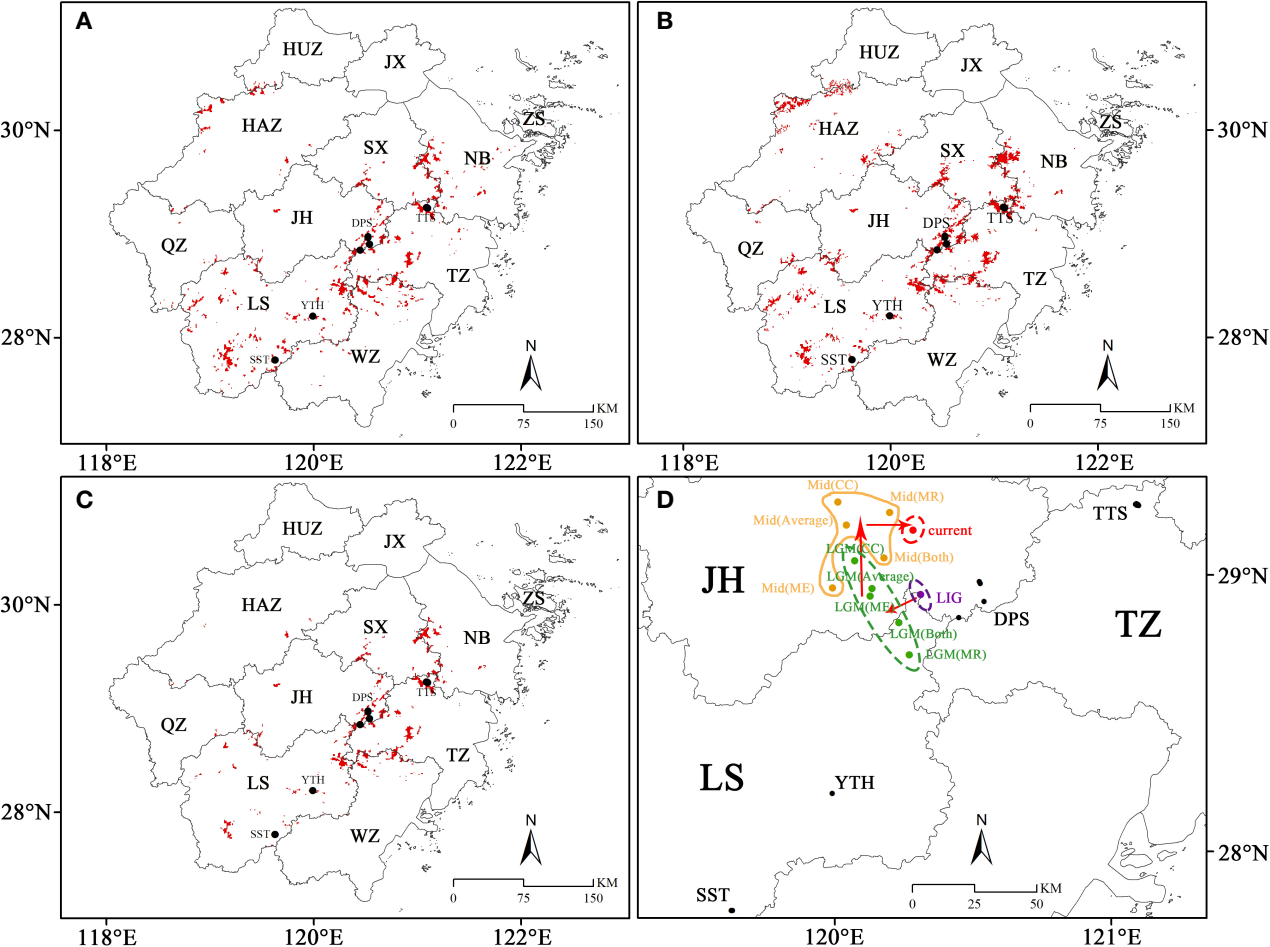
The SDAs (suitable distribution areas) and centroid migration of *C. tientaiensis* in Zhejiang. **(A, B)** The public regions that were all SDAs in three GCMs (Global Climate Models) during the LGM (Last Glacial Maximum, about 21,000 years BP) and Mid (Mid-Holocene, about 6,000 years BP) periods, respectively. **(C)** The public regions that were all SDAs in the paleo and current four periods. **(D)** The migration of the centroid under different periods. HUZ, Huzhou city; JX, Jiaxing city; HAZ, Hangzhou city; SX, Shaoxing city; NB, Ningbo city; JH, Jinhua city; QZ, Quzhou city; TZ, Taizhou city; WZ, Wenzhou city; LS, Lishui city; TTS, Tiantai Mountain, Taizhou city; DPS, Dapan Mountain, Jinhua city; YTH, Yangtianhe, Lishui city; SST, Shangshantou, Lishui city; LGM: Last Glacial Maximum, about 21,000 years BP; Mid: Mid-Holocene, about 6,000 years BP; CC (Community Climate System Model version 4, CCSM4); MR (the Model for Interdisciplinary Research on Climate, Earth System Model, MIROC-ESM); ME (the Max Planck Institute for Meteorology Earth System Model, MPI-ESM-P).

In Zhejiang, the regions where were all SDAs in four periods were mainly located around DPS and TTS in eastern Zhejiang, while only sporadically distributed in southern Zhejiang (**Figure 9C**). Moreover, the four extant natural populations were suitable distribution areas during the four periods. Although *C. tientaiensis* has not yet been found in Kuocang Mountains at the border of Taizhou and Wenzhou and Siming Mountains at the border of Shaoxing and Ningbo in eastern Zhejiang, there were all suitable distribution areas for *C. tientaiensis* during these four periods (**Figure 9C**). In Zhejiang, the mean center of the suitable distribution areas was primarily found in the west or northwest of DPS (**Figure 9D**). It was located in the west of DPS during the LIG, migrated further to its west during the LGM, migrated to the north during the Mid, and migrated to the east during the current climate scenario (**Figure 9D**).

## 4 Discussion

### 4.1 Genetic variation of *C. tientaiensis*

It is discovered that the haplotype diversity (*H*_d_) of *C. tientaiensis* was 0.6825. Petit et al. (2005) calculated that the average chloroplast variation of 170 species was 0.67, so *C. tientaiensis* had moderate genetic diversity. The majority of angiosperms inherited their cpDNA matrilineally (Hu et al., 2008; Zhang and Sodmergen, 2010), which was done through seeds. Previous research has shown that the male flowers of *C. tientaiensis* begin to blossom earlier than the female flowers, and higher air humidity and rainy weather may have adverse effects on pollination (Zhang et al., 2016). These factors have a negative impact on the fruiting of this species, and the topography of mountains and hills in Zhejiang also restricted the dispersal of pollen and seeds, which hindered gene flow to a certain extent, leading to a certain degree of genetic differentiation. Consequently, its cpDNA sequences were able to preserve a certain degree of genetic variation.

The cpDNA can be used to analyze the genetic diversity of *C. tientaiensis* to a significant extent, but due to the limited sequence length, it was difficult to comprehensively evaluate its genetic variation. At the same time, because there has been extensive hybridization and introgression among the *Carpinus* species (Wang et al., 2022), which may lead to inconsistency between the results of nuclear gene and cpDNA. Therefore, it is critical to detect the genetic variation of *C. tientaiensis* comprehensively. At the same time, AFLP markers can cover the entire genome without the need for obtaining the DNA sequence beforehand (Vos et al., 1995). The *PPBs* can indicate the degree of genetic diversity of species. This study indicated that *PPBs* of *C. tientaiensis*, at about 76.16% at the species level, were substantially higher than kiwifruit (Zhang et al., 2018) and the endangered species *Bretschneidera sinensis* (Hu et al., 2017). Hamrick et al. (1992) found that the *PPBs* of woody species were about 77% in his analysis of the genetic diversity of 662 species. Accordingly, AFLP markers can effectively reveal the genetic variation of *C. tientaiensis*.

Nybom (2004) examined the genetic diversity of plants using several molecular markers and discovered that the average genetic diversity of perennial plants was 0.25 whereas the regional distribution species was 0.21. According to AFLP markers, the total genetic diversity (*Ht*) of *C. tientaiensis* was approximately 0.1516, which was comparable to the endangered plants *Camellia nitidisima* (Li et al., 2019) and *Horsfieldia pandurifolia* (Mao et al., 2020) but lower than *B. sinensis* (Hu et al., 2017). The different ripening time of pistils and stamens and the windy and rainy climate conditions in Zhejiang made it difficult for *C. tientaiensis* to bear fruit, which hindered the regeneration of the community. Widespread hybridization and introgression caused genetic drift and allele loss. Moreover, due to the destruction of the wild community during the construction of tourist sites and reservoirs, *C. tientaiensis* has low genetic diversity.

### 4.2 Genetic structure and population differentiation of *C. tientaiensis*

Wright (1978) proposed the genetic differentiation standard according to genetic differentiation coefficient (Gst) and stated that there was virtually little genetic differentiation when *Gst* ≤ 0.05; there was genetic differentiation when 0.05 < *Gst* ≤ 0.15; there was a large genetic differentiation when 0.15 < *Gst* ≤ 0.25; and there was significant genetic differentiation when *Gst* > 0.25. Previous study found that the average *Gst* of endangered species was about 0.141 (Hamrick et al., 1990). Petit et al. (2005) calculated the *Gst* of the cpDNA of 124 angiosperms was 0.637. This study discovered that the *Gst* of *C. tientaiensis*, based on cpDNA and AFLP markers, was 0.2333 and 0.714, respectively, which indicated that there was large genetic differentiation between populations. Pollen and seeds from plants can only travel a limited amount of distance, and the terrain of Zhejiang, which includes mountains and hills, as well as long-distance geographic barriers, would restrict the transmission of seeds and pollen. This has promoted the genetic variation to some extent, contributing to its high genetic differentiation.

Zhejiang, a province in eastern China, has a complicated terrain, with TTS and DPS located in the hilly areas in eastern Zhejiang, and YTH and SST located in the mountainous areas in southern Zhejiang. The population differentiation between the southern geographical group and northern geographical group of *C. tientaiensis* has been caused by the restricted gene flow due to the crisscross terrain of hills and mountains in Zhejiang, and the unique genotype can be maintained among the geographical groups. There were 5 private haplotypes and a much higher haplotype diversity (*H*_d_) in TTS population than other populations. The SST population has the greatest number of wild individuals and the highest genetic diversity according to AFLP markers. The primitive communities tend to have more unique haplotypes, and their genetic diversity is often higher than that of communities with migration and diffusion due to the “founder effect” (Mayr, 1942; Brooks and Yamamoto, 2021). As a result, TTS and SST were presumably the original communities and origins of *C. tientaiensis*.

### 4.3 Effects of environmental on genetic differentiation and distribution of *C. tientaiensis*

The significant topographic and climatic differences in China greatly affected the population differentiation and spatial distribution of genetic variation of plants in China (Zhang et al., 2013a; Zhang et al., 2013b; Shi et al., 2014; Sun et al., 2016). This study demonstrated that topographic and climatic differences on the regional scale still had a profound impact on the population differentiation of plants. The natural populations of *C. tientaiensis* may have experienced long-term isolation by distance due to the complex landscapes of hills, mountains, and plains in Zhejiang. This study discovered that genetic diversity and differentiation of *C. tientaiensis* may be significantly impacted by extreme temperature, rainfall, and other climatic factors. As a typical temperate tree species, extremely cold and high temperature, and exceptionally dry weather may affect the survival of *C. tientaiensis*. In addition, its pollination and fruiting may be significantly impacted by temperature and rainfall (Zhang et al., 2016), and these variables may cause isolation by distance among populations.

Genetic differentiation was frequently a result of isolation by distance and environment (Mayr, 1947). Isolated populations may occupy unique niches because they progressively adapt to the local environment (Rundle and Nosil, 2005). For this reason, niche differentiation can lead to genetic differentiation between species or populations. Although there was no glacier in eastern Zhejiang during the Quaternary ice age (Sang et al., 2011), the climate change in Quaternary had significantly affected the species spatial distribution and differentiation in China (Qiu et al., 2011). In this study, it can be found that previous climate change had a prominence effect on distribution of *C. tientaiensis*, and suitable distribution areas in southern Zhejiang were repeatedly impacted by climate change based on MaxEnt model. At the same time, the suitable distribution areas in Zhejiang were primarily distributed in eastern and southern Zhejiang, with obvious niche differentiation. As a result, historical climate change might have encouraged the genetic differentiation of *C. tientaiensis*.

### 4.4 Protection and management for *C. tientaiensis*

*C. tientaiensis* was sensitive to climate change, and paleo climate change has significantly affected its spatial distribution and differentiation. The Kuocang and Siming Mountains were suitable distribution areas of *C. tientaiensis* during the current and three paleo climate periods, and should be key areas for future field research, as there may be undiscovered wild individuals here. Research has found that future climate change will seriously affect its survival and distribution, with a sharp decrease in its suitable distribution and core suitable areas, especially in southern Zhejiang, where the suitable distribution areas were almost completely lost (Zhao et al., 2020). Therefore, under the future climate change, it is necessary to strengthen the protection and management of SST population in southern Zhejiang to prevent the population from disappearing. At the same time, as local tourism has expanded recently, SST has attracted an increasing number of visitors, which ought to raise some alarms for us. Conducting artificial assisted breeding in SST population has special advantages, as SST is the population with the largest known wild population of *C. tientaiensis*, and it is located in a remote and rarely traveled area. This study proposed to establish a nature reserve in SST, carry out germplasm resource collection and artificial breeding, and conduct scientific research to analyze its survival and adaptation mechanisms.

This study indicated that SST and TTS populations have high genetic diversity, and they were also the populations with the most individuals. Among them, TTS was a well-known scenic spot in China, and numerous tourists have brought many negative impacts on the survival of *C. tientaiensis*. To a certain extent, the cultivation management and artificial breeding of the management department of TTS have also protected the survival of *C. tientaiensis*. Furthermore, the local management agency department keep enhancing conservation management, safeguard original habitat, and establish core reserves to reduce tourists. The YTH population had the fewest individuals and the least genetic diversity. In this region, the building of dams and reservoirs were likely to greatly damage the community of *C. tientaiensis*, leading to a sharp decrease in population size. Therefore, it was essential to conduct artificial breeding to increase population size and prevent the extinction of the existing individuals. The DPS has not yet undergone tourism development, but there were very few individuals and the distance between individuals was very far. Therefore, artificial breeding and cultivation should be carried out for DPS population to resist the adverse conditions of natural breeding, and conservation management should be strengthened in the original habitats to expand the number of individuals.

## 5 Conclusions

*C. tientaiensis* has significant economic and ecological value. Scientifically analyzing its genetic diversity and differentiation and suggesting appropriate protection methods have significant implications for enhancing resource preservation and species usage. This study found that *C. tientaiensis* had a relatively low genetic diversity, and TTS and SST populations may not only be the genesis and cradle of *C. tientaiensis*, but also its current hub of genetic diversity. There was genetic differentiation between populations, and it was extremely correlated with geographical and bioclimatic factors. The populations located in the hilly areas of eastern Zhejiang and the mountainous areas of southern Zhejiang have formed two distinct genetic branches, with DPS and YTH populations serving as the link for gene exchange between these two genetic branches. The genetic structure of *C. tientaiensis* was shaped by both the topography and climatic conditions of the mountains and hills in East China.

## Data availability statement

The original contributions presented in the study are included in the article/**Supplementary Material**, further inquiries can be directed to the corresponding author.

## Author contributions

RZ: Conceptualization, Data curation, Formal analysis, Funding acquisition, Investigation, Methodology, Project administration, Writing – original draft, Writing – review & editing. QH: Formal analysis, Investigation, Writing – review & editing. XC: Formal analysis, Writing – review & editing. AH: Investigation, Writing – review & editing. YZ: Funding acquisition, Writing – review & editing. ZZ: Funding acquisition, Project administration, Supervision, Writing – review & editing.
