# Supplementary material for: Regional environmental differences significantly affect the genetic structure and genetic differentiation of Carpinus tientaiensis Cheng, an endemic and extremely endangered species from China
Source: Front Plant Sci. 2024 Feb 9;15:1277173. doi: 10.3389/fpls.2024.1277173 (PMC10885731; doi:10.3389/fpls.2024.1277173)
Supplement: Supplementary file 1 [file DataSheet_1.pdf]

Supplementary Material

Regional environmental differences significantly affect the genetic structure and genetic differentiation of *Carpinus tientaiensis* Cheng, an endemic and extremely endangered species from China

Runan Zhao [1,2], Qianqian He [3], Xiaojie Chu [4], Anguo He [5], Yuanlan Zhang [2,6*], and Zunling Zhu [1,2,7,*]

[1] College of Landscape Architecture, Nanjing Forestry University, Nanjing, China

[2] Co-Innovation Center for Sustainable Forestry in Southern China, Nanjing Forestry University, Nanjing, China

[3] Research Center for Urban and Rural Living Environment, Zhijiang College of Zhejiang University of Technology, Shaoxing, China

[4] College of Life Sciences, Zhejiang Normal University, Jinhua, China

[5] Administration of Zhejiang Dapanshan National Nature Reserve, Pan'an, China

[6] College of Life Sciences, Nanjing Forestry University, Nanjing, China

[7] Jinpu Research Institute, Nanjing Forestry University, Nanjing, China

*** Correspondence:**

zhuzunling@njfu.edu.cn (Zunling Zhu); nlzyl2020@163.com (Yuanlan Zhang)

Supplementary Table S1. The geographical location and number of individuals of *C. tientaiensis*.

Population	Geographical location	Longitude	Latitude	Altitude (m)	Sample sizes
DPS	Dapanshan National Nature Reserve, Pan'an County, Jinhua City, China	120.5218	28.9708	1138	5
TTS	Tiantai Mountain, Tiantai County, Tianzhou City, China	121.0917	29.2568	901	17
YTH	Yangtianhe, Qingtian County, Lishui City, China	119.9907	28.2084	1249	2
SST	Shangshantou, Jingning She Autonomous County, Lishui City, China	119.6320	27.7823	1506	29

Supplementary Table S2. Reaction system.

Reagent	Contrast group	Treatment group
Control template DNA	4 µl	-
Template DNA	-	4 µl
Adapter	1 µl	1 µl
*Eco*R I/*Mse* I	2 µl	2 µl
10×Reaction buffer	2.5 µl	2.5 µl
10mM ATP	2.5 µl	2.5 µl
T4 Ligase	1 µl	1 µl
AFLP-Water	7 µl	7 µl
Total	20 µl	20 µl

Supplementary Table S3. Joint sequences.

Connector	Sequence 1 (5’-3’)	Sequence 2 (5’-3’)
*Eco*R I	CTCGTAGACTGCGTACC	AATTGGTACGCAGTCTAC
Mse I	GACGATGAGTCCTGAG	TACTCAGGACTCAT

Supplementary Table S4. Pre-amplification reaction system.

Reagent	Volume
Template DNA	2 µl
Pre-ampmix	1 µl
dNTPs	0.5 µl
10×PCR buffer	2.5 µl
Taq DNA polymease	0.5 µl
ddH_2O	18.5 µl
Total	20 µl

Supplementary Table S5. Joint sequences.

Connector	Sequence 1(5'-3')	Sequence 2(5'-3')
*Eco*R I	CTCGTAGACTGCGTACC	AATTGGTACGCAGTCTAC
Mse I	GACGATGAGTCCTGAG	TACTCAGGACTCAT

Supplementary Table S6. AFLP primer sequences.

***Eco*R I (5ng/µl)**		***Mse* I (30ng/µl)**	
**Code**	**Primers sequence (5'-3')**	**Code**	**Primers sequence (5'-3')**
E-AAC	GACTGCGTACCAATTCAAC	*M-CAA*	GATGAGTCCTGAGTAACAA
E-AAG	GACTGCGTACCAATTCAAG	*M-CAC*	GATGAGTCCTGAGTAACAC
E-ACA	GACTGCGTACCAATTCACA	*M-CAG*	GATGAGTCCTGAGTAACAG
E-ACT	GACTGCGTACCAATTCACT	*M-CAT*	GATGAGTCCTGAGTAACAT
E-ACC	GACTGCGTACCAATTCACC	*M-CTA*	GATGAGTCCTGAGTAACTA
E-ACG	GACTGCGTACCAATTCACG	*M-CTC*	GATGAGTCCTGAGTAACTC
E-AGC	GACTGCGTACCAATTCAGC	*M-CTG*	GATGAGTCCTGAGTAACTG
E-AGG	GACTGCGTACCAATTCAGG	*M-CTT*	GATGAGTCCTGAGTAACTT

Supplementary Table S7. Amplification reaction system.

Reagent	Volume
Pre amplified samples after dilution	2 µl
10×PCR buffer	2.5 µl
dNTPs	0.5 µl
*Eco*R I primer	1 µl
Mse I primer	1 µl
Taq enzyme	0.5 µl
ddH_2O	17.5 µl
Total	25 µl

Supplementary Table S8. Primer combinations for amplification.

Primer pairs	*Eco*R I primers (5'-3')	*Mse* I primers (5'-3')
E-ACA/M-CAG	GACTGCGTACCAATTCACA	GATGAGTCCTGAGTAACAG
E-ACC/M-CTC	GACTGCGTACCAATTCACC	GATGAGTCCTGAGTAACTC
E-ACG/M-CAA	GACTGCGTACCAATTCACG	GATGAGTCCTGAGTAACAA
E-ACG/M-CAT	GACTGCGTACCAATTCACG	GATGAGTCCTGAGTAACAT
E-AGC/M-CAC	GACTGCGTACCAATTCAGC	GATGAGTCCTGAGTAACAC
E-AGC/M-CAG	GACTGCGTACCAATTCAGC	GATGAGTCCTGAGTAACAG
E-AGC/M-CTC	GACTGCGTACCAATTCAGC	GATGAGTCCTGAGTAACTC
E-AGC/M-CTG	GACTGCGTACCAATTCAGC	GATGAGTCCTGAGTAACTG

Supplementary Table S9. Primers and annealing temperature of cpDNA sequences.

Primers	Primer sequences (5'-3')	T_A (℃)	Length (bp)	References
*trn*L-*trn*F	F: AAAATCGTGAGGGTTCAAGTC<br>R: GATTTGAACTGGTGACACGAG	56	452	Sang *et al*., 1997
*trn*G	F: GGTAAAAGTGTGATTCGTTC<br>R: GTTTCATTCGGCTCCTTTAT	58	603	Nishizawa *et al*., 2000
*rps*16	F: GTGGTAGAAAGCAACGTGCGACTT<br>R: GGTTTAGACATTACTTCGTTGA	56	803	Oxelman *et al*., 1997
*atp*B-*rbc*L	F: TAGTTTCTGTTTGTGGTGACAT<br>R: AAGTAGTAGGATTGGTTCTCAT	55	718	Okaura *et al*., 2007
*psb*A-*trn*H	F: ACGGGAATTGAACCCGCGCA<br>R: TATTATTAACCGTGCTAACC	57	514	Okaura *et al*., 2007
*pet*B-*pet*D	F: CAATCCACTTTGACTCGTTTT<br>R: GGTTCACCAATCATTGATGGTTC	60	612	Dong *et al*., 2012
*trn*S(GCU)-*trn*G(UCC)	F: GCCGCTTTAGTCCACTCAGC<br>R: GAACGAATCACACTTTTACCAC	60	607	Hamilton, 1999
*trn*S(GCU)-*trn*T(GGU)	F: GAGATGGCCGAGTGGTTGAA<br>R: CCCGCTTAGCTCAGAGGTTAGAG	60	902	Kanno *et al*., 2004

References

Dong WP, Liu J, Yu J, *et al*., 2012. Highly Variable Chloroplast Markers for Evaluating Plant Phylogeny at Low Taxonomic Levels and for DNA Barcoding. Plos one. 7: e35071.

Hamilton MB, 1999. Four primer pairs for the amplification of chloroplast intergenic regions with intraspecific variation. Mol. Ecol. 8(3): 521-523. In: 1999. Primer Notes. Mol. Ecol. 8: 513-525.

Kanno M, Yokoyama J, Suyama Y, *et al*., 2004. Geographical distribution of two haplotypes of chloroplast DNA in four oak species (*Quercus*) in Japan. J. Plant Res. 117: 311-317.

Nishizawa T, Watano Y, 2000. Primer pairs suitable for PCR-SSCP analysis of chloroplast DNA in angiosperms. J. Phytogeogr. Taxon. 48: 63-66.

Okaura T, Quang ND, Ubukata M, *et al*., 2007. Phylogeographic structure and late Quaternary population history of the Japanese oak *Quercus mongolica* var. *crispula* and related species revealed by chloroplast DNA variation. Genes Genet. Syst. 82: 465-477.

Oxelman B, Lidén M, Berglund D, 1997. Chloroplast rps16 intron phylogeny of the tribe Sileneae (Caryophyllaceae). Plant Syst. Evol. 206: 393-410.

Sang T, Crawford DJ, Stuessy TF, 1997. Chloroplast DNA phylogeny, reticulate evolution and biogeography of *Paeonia* (Paeoniaceae). Am. J. Bot. 84: 1120-1136.

Supplementary Table S10. The 19 bioclimatic variables and jackknife analysis results of MaxEnt model.

Variable	Variable description	Unit	Contribution Rate (%)
bio11	Mean temperature of coldest quarter	℃	28.1
bio10	Mean temperature of warmest quarter	℃	21.4
bio17	Precipitation of the driest quarter	mm	21.1
bio03	Isothermality (bio2 /bio7 × 100)	%	12.6
bio08	Mean temperature of wettest quarter	℃	6.7
bio15	Coefficient of variation (CV) of precipitation seasonality	%	3.8
bio16	Precipitation of wettest quarter	mm	2.2
bio05	Max temperature of the warmest month	℃	1.0
bio19	Precipitation of the coldest quarter	mm	0.7
bio12	Annual precipitation	mm	0.7
bio09	Mean temperature of driest quarter	℃	0.6
bio06	Min temperature of coldest month	℃	0.5
bio01	Annual mean temperature	℃	0.3
bio18	Precipitation of the coldest quarter	mm	0.1
bio04	Standard deviation (SD) of temperature seasonality	%	0.1
bio14	Precipitation of the driest month	mm	0
bio13	Precipitation of wettest month	mm	0
bio02	Mean diurnal temperature range	℃	0
bio07	Range of annual temperature	℃	0

Supplementary Table S11. The 12 effective distribution data used in the MaxEnt model.

Species	Longitude	Latitude	Location
C. tientaiensis	121.09915	29.25046	Tianzhou City, Zhejiang Province, China
C. tientaiensis	121.09314	29.25175	Tianzhou City, Zhejiang Province, China
C. tientaiensis	121.08853	29.25437	Tianzhou City, Zhejiang Province, China
C. tientaiensis	121.09706	29.25392	Tianzhou City, Zhejiang Province, China
C. tientaiensis	121.09170	29.25683	Tianzhou City, Zhejiang Province, China
C. tientaiensis	120.52585	28.96782	Jinhua City, Zhejiang Province, China
C. tientaiensis	120.44910	28.84516	Jinhua City, Zhejiang Province, China
C. tientaiensis	120.52304	28.97513	Jinhua City, Zhejiang Province, China
C. tientaiensis	120.54056	28.90278	Jinhua City, Zhejiang Province, China
C. tientaiensis	119.99075	28.20843	Lishui City, Zhejiang Province, China
C. tientaiensis	119.63037	27.78506	Lishui City, Zhejiang Province, China
C. tientaiensis	119.62528	27.78528	Lishui City, Zhejiang Province, China

Supplementary Table S12. The correlation coefficient between the 19 bioclimatic variables.

	bio01	bio02	bio03	bio04	bio05	bio06	bio07	bio08	bio09	bio10	bio11	bio12	bio13	bio14	bio15	bio16	bio17	bio18	bio19
**bio01**	1	-0.252	0.543	-0.605	0.605	0.919	-0.600	0.586	0.922	0.761	0.947	0.448	0.482	0.172	0.296	0.536	0.165	0.500	0.255
**bio02**	-0.252	1	0.080	0.486	0.197	-0.497	0.610	-0.031	-0.359	0.062	-0.384	-0.371	-0.301	-0.188	-0.135	-0.358	-0.117	-0.428	-0.095
**bio03**	0.543	0.080	1	-0.814	-0.145	0.648	-0.734	0.253	0.620	0.009	0.703	0.443	0.560	-0.114	0.577	0.590	-0.126	0.654	-0.002
**bio04**	-0.605	0.486	-0.814	1	0.237	-0.846	0.983	-0.166	-0.748	0.054	-0.828	-0.610	-0.640	-0.030	-0.550	-0.709	0.026	-0.767	-0.086
**bio05**	0.605	0.197	-0.145	0.237	1	0.288	0.253	0.453	0.385	0.956	0.332	-0.039	-0.056	0.194	-0.208	-0.044	0.241	-0.195	0.253
**bio06**	0.919	-0.497	0.648	-0.846	0.288	1	-0.854	0.449	0.936	0.469	0.990	0.563	0.587	0.139	0.430	0.659	0.101	0.665	0.198
**bio07**	-0.600	0.610	-0.734	0.983	0.253	-0.854	1	-0.207	-0.736	0.046	-0.819	-0.590	-0.624	-0.035	-0.547	-0.689	0.029	-0.778	-0.062
**bio08**	0.586	-0.031	0.253	-0.166	0.453	0.449	-0.207	1	0.388	0.598	0.473	-0.157	-0.009	-0.326	0.416	-0.003	-0.348	0.237	-0.337
**bio09**	0.922	-0.359	0.620	-0.748	0.385	0.936	-0.736	0.388	1	0.548	0.953	0.602	0.605	0.270	0.285	0.660	0.250	0.587	0.381
**bio10**	0.761	0.062	0.009	0.054	0.956	0.469	0.046	0.598	0.548	1	0.515	0.065	0.078	0.189	-0.079	0.092	0.225	-0.001	0.244
**bio11**	0.947	-0.384	0.703	-0.828	0.332	0.990	-0.819	0.473	0.953	0.515	1	0.565	0.597	0.142	0.419	0.662	0.114	0.657	0.222
**bio12**	0.448	-0.371	0.443	-0.610	-0.039	0.563	-0.590	-0.157	0.602	0.065	0.565	1	0.899	0.629	0.002	0.922	0.625	0.765	0.690
**bio13**	0.482	-0.301	0.560	-0.640	-0.056	0.587	-0.624	-0.009	0.605	0.078	0.597	0.899	1	0.375	0.334	0.972	0.376	0.873	0.461
**bio14**	0.172	-0.188	-0.114	-0.030	0.194	0.139	-0.035	-0.326	0.270	0.189	0.142	0.629	0.375	1	-0.618	0.389	0.976	0.099	0.941
**bio15**	0.296	-0.135	0.577	-0.550	-0.208	0.430	-0.547	0.416	0.285	-0.079	0.419	0.002	0.334	-0.618	1	0.337	-0.648	0.534	-0.565
**bio16**	0.536	-0.358	0.590	-0.709	-0.044	0.659	-0.689	-0.003	0.660	0.092	0.662	0.922	0.972	0.389	0.337	1	0.386	0.889	0.468
**bio17**	0.165	-0.117	-0.126	0.026	0.241	0.101	0.029	-0.348	0.250	0.225	0.114	0.625	0.376	0.976	-0.648	0.386	1	0.079	0.957
**bio18**	0.500	-0.428	0.654	-0.767	-0.195	0.665	-0.778	0.237	0.587	-0.001	0.657	0.765	0.873	0.099	0.534	0.889	0.079	1	0.134
**bio19**	0.255	-0.095	-0.002	-0.086	0.253	0.198	-0.062	-0.337	0.381	0.244	0.222	0.690	0.461	0.941	-0.565	0.468	0.957	0.134	1

Supplementary Table S13. Contribution rate of the 9 bioclimatic variables in the MaxEnt model in current period.

Variable	Variable description	Unit	Contribution Rate (%)
bio11	Mean temperature of coldest quarter	℃	25.2
bio10	Mean temperature of warmest quarter	℃	23.5
bio17	Precipitation of the driest quarter	mm	20.0
bio03	Isothermality (bio2 /bio7 × 100)	%	13.9
bio08	Mean temperature of wettest quarter	℃	11.0
bio15	CV of precipitation seasonality	%	2.7
bio16	Precipitation of wettest quarter	mm	2.1
bio04	SD of temperature seasonality	%	1.4
bio02	Mean diurnal temperature range	℃	0.1

Supplementary Table S14. The area under the curve (AUC), minimum training presence area, maximum test sensitivity plus specificity area, and equate entropy of thresholded and original distributions area of MaxEnt models under different regularization multipliers.

Regularization multipliers	Training AUC	Test AUC	Minimum training presence area	Maximum test sensitivity plus specificity area	Equate entropy of thresholded and original distributions area
0.25	0.9994	0.9993	0.0014	0.0007	0.0026
0.3	0.9994	0.9993	0.0014	0.0007	0.0028
0.4	0.9994	0.9993	0.0015	0.0008	0.0033
0.5	0.9994	0.9993	0.0015	0.0008	0.0037
0.6	0.9993	0.9992	0.0015	0.0008	0.0042
0.7	0.9993	0.9992	0.0016	0.0009	0.0046
0.75	0.9993	0.9992	0.0016	0.0009	0.0049
1.0	0.9993	0.9991	0.0018	0.001	0.0062
1.25	0.9991	0.9991	0.0022	0.0011	0.0076
1.5	0.9991	0.999	0.0024	0.0011	0.0091
1.75	0.999	0.9989	0.0028	0.0013	0.0106
2.0	0.9988	0.9988	0.0035	0.0015	0.0122

Supplementary Table S15. The number of loci amplified by each primer pair.

Primer pairs	DPS		TTS		YTH		SST		Total		Loci
	N	*PPBs*	*N*	*PPBs*	*N*	*PPBs*	*N*	*PPBs*	*N*	*PPBs*	
E-ACA/M-CAG	80	37.04%	122	56.48%	40	18.52%	129	59.72%	162	75%	216
E-ACC/M-CTC	81	37.50%	141	65.28%	32	14.81%	150	69.44%	178	82.41%	216
E-ACG/M-CAA	119	55.09%	141	65.28%	44	20.37%	159	73.61%	178	82.41%	216
E-ACG/M-CAT	63	29.17%	104	48.15%	29	13.43%	129	59.72%	153	70.83%	216
E-AGC/M-CAC	81	37.50%	115	53.24%	24	11.11%	143	66.20%	156	72.22%	216
E-AGC/M-CAG	72	33.33%	111	51.39%	23	10.65%	118	54.63%	155	71.76%	216
E-AGC/M-CTC	105	48.61%	144	66.67%	61	28.24%	139	64.35%	179	82.87%	216
E-AGC/M-CTG	68	31.48%	108	50.00%	25	11.57%	130	60.19%	155	71.76%	216
Mean	84	38.72%	123	57.06%	35	16.09%	137	63.48%	165	76.16%	216
Total	669	38.72%	986	57.06%	278	16.09%	1097	63.48%	1316	76.16%	1728

Supplementary Table S16. Genetic diversity of *C. tientaiensis.*

Primer pairs	Population	*Na*	*Ne*	*H*	*I*
E-ACA/M-CAG	DPS	1.3704±0.4840	1.2008±0.3185	0.1214±0.1759	0.1854±0.2578
	TTS	1.5648±0.4969	1.2317±0.3134	0.1450±0.1728	0.2290±0.2502
	YTH	1.1852±0.3894	1.1309±0.2753	0.0767±0.1613	0.1120±0.2354
	SST	1.5972±0.4916	1.2249±0.3124	0.1403±0.1727	0.2224±0.2485
	Total	1.7500±0.4340	1.2449±0.3140	0.1548±0.1695	0.2495±0.2400
E-ACC/M-CTC	DPS	1.3750±0.4852	1.2103±0.3291	0.1257±0.1801	0.1908±0.2626
	TTS	1.6528±0.4772	1.2449±0.3204	0.1533±0.1722	0.2450±0.2446
	YTH	1.1481±0.3561	1.1048±0.2518	0.0614±0.1475	0.0896±0.2153
	SST	1.6944±0.4617	1.2384±0.3098	0.1512±0.1684	0.2436±0.2399
	Total	1.8241±0.3816	1.2624±0.3146	0.1668±0.1677	0.2708±0.2334
E-ACG/M-CAA	DPS	1.5509±0.4986	1.3114±0.3616	0.1853±0.1930	0.2809±0.2772
	TTS	1.6528±0.4772	1.2794±0.3380	0.1717±0.1800	0.2696±0.2550
	YTH	1.2037±0.4037	1.1440±0.2854	0.0844±0.1672	0.1232±0.2441
	SST	1.7361±0.4418	1.2681±0.3139	0.1709±0.1672	0.2751±0.2363
	Total	1.8241±0.3816	1.2909±0.3162	0.1853±0.1672	0.2980±0.2328
E-ACG/M-CAT	DPS	1.2917±0.4556	1.1349±0.2640	0.0850±0.1488	0.1335±0.2226
	TTS	1.4815±0.5008	1.1879±0.2972	0.1180±0.1639	0.1879±0.2392
	YTH	1.1343±0.3417	1.0949±0.2416	0.0556±0.1415	0.0812±0.2066
	SST	1.5972±0.4916	1.1893±0.2920	0.1203±0.1611	0.1956±0.2321
	Total	1.7083±0.4556	1.2075±0.2981	0.1325±0.1625	0.2165±0.2321
E-AGC/M-CAC	DPS	1.3750±0.4852	1.1803±0.2913	0.1131±0.1642	0.1763±0.2440
	TTS	1.5324±0.5001	1.1832±0.2844	0.1176±0.1579	0.1910±0.2302
	YTH	1.1111±0.3150	1.0786±0.2227	0.0460±0.1305	0.0672±0.1905
	SST	1.6620±0.4741	1.2158±0.3022	0.1376±0.1640	0.2237±0.2343
	Total	1.7222±0.4489	1.2154±0.2939	0.1399±0.1574	0.2316±0.2234

Supplementary Table S16. Cont.

Primer pairs	Population	*Na*	*Ne*	*H*	*I*
E-AGC/M-CAG	DPS	1.3333±0.4725	1.1708±0.2957	0.1049±0.1654	0.1617±0.2442
	TTS	1.5139±0.5010	1.1869±0.2885	0.1195±0.1594	0.1929±0.2333
	YTH	1.1065±0.3092	1.0753±0.2186	0.0441±0.1281	0.0644±0.1870
	SST	1.5463±0.4990	1.1751±0.2734	0.1140±0.1536	0.1865±0.2257
	Total	1.7176±0.4512	1.1915±0.2714	0.1268±0.1521	0.2111±0.2199
E-AGC/M-CTC	DPS	1.4861±0.5010	1.2573±0.3337	0.1569±0.1834	0.2406±0.2674
	TTS	1.6667±0.4725	1.2530±0.3014	0.1627±0.1667	0.2607±0.2401
	YTH	1.2824±0.4512	1.1997±0.3191	0.1170±0.1869	0.1708±0.2729
	SST	1.6435±0.4801	1.2083±0.2927	0.1341±0.1610	0.2186±0.2322
	Total	1.8287±0.3776	1.2503±0.2987	0.1625±0.1613	0.2661±0.2268
E-AGC/M-CTG	DPS	1.3148±0.4655	1.1650±0.2937	0.1009±0.1646	0.1549±0.2429
	TTS	1.5000±0.5012	1.1776±0.2827	0.1136±0.1580	0.1835±0.2311
	YTH	1.1157±0.3207	1.0818±0.2267	0.0479±0.1328	0.0700±0.1939
	SST	1.6019±0.4907	1.2016±0.2877	0.1299±0.1614	0.2103±0.2349
	Total	1.7176±0.4512	1.2016±0.2782	0.1324±0.1559	0.2186±0.2249
Mean	DPS	1.3872±0.0825	1.2039±0.0529	0.1242±0.0304	0.1905±0.0451
	TTS	1.5705±0.0711	1.2181±0.0364	0.1377±0.0217	0.2200±0.033
	YTH	1.1609±0.0565	1.1138±0.0399	0.0666±0.0234	0.0973±0.0342
	SST	1.6348±0.0574	1.2152±0.0273	0.1373±0.0167	0.2220±0.0260
	Total	1.7616±0.0509	1.2331±0.0323	0.1501±0.0192	0.2453±0.0291

Supplementary Table S17. Genetic diversity, genetic differentiation coefficient, and gene flow of *C. tientaiensis.*

Primer pairs	*Ht*	*Hs*	*Gst*	*Nm*
E-ACA/ M-CAG	0.1513±0.0269	0.1209±0.0163	0.2012	1.9849
E-ACC/ M-CTC	0.1647±0.0292	0.1229±0.0151	0.2538	1.4697
E-ACG/M-CAA	0.1944±0.0313	0.1531±0.0184	0.2126	1.8515
E-ACG/M-CAT	0.1262±0.0261	0.0947±0.0144	0.2491	1.5070
E-AGC/M-CAC	0.1384±0.0262	0.1036±0.0132	0.2519	1.4848
E-AGC/M-CAG	0.1273±0.0254	0.0956±0.0129	0.2489	1.5087
E-AGC/M-CTC	0.1847±0.0294	0.1427±0.0175	0.2274	1.6988
E-AGC/M-CTG	0.1260±0.0249	0.0981±0.0138	0.2217	1.7550
Mean	0.1516±0.0254	0.1165±0.0209	0.2333	1.6576

Supplementary Table S18. Analysis of molecular variance (AMOVA).

Source of variation		Quadratic sum	Variance	Percentage (%)	Fixed coefficient
AFLP	Among populations	2098.459	26.6809	15.47503	0.15475 ($P < 0.00001$)
	Within populations	14864.635	145.73172	84.52497	
	Total	16963.094	172.41262		
cpDNA	Among populations	57.457	1.83486	85.55536	0.85555 ($P < 0.00001$)
	Within populations	14.870	0.30979	14.44464	
	Total	72.327	2.14465		

Supplementary Table S19. Nine haplotypes identified based on 2 singleton variable sites and 8 parsimony information sites.

Haplotypes	***trn*L-*trn*F**		***trn*G**			***psb*A-*trn*H**					Number
	**348**	**349**	**605**	**902**	**1037**	**1279**	**1396**	**1534**	**1540**	**1533**	
H1	T	T	-	A	T	C	T	G	T	T	28
H2	T	T	-	A	T	C	T	G	G	T	1
H3	T	T	-	A	G	C	T	G	T	T	1
H4	T	-	A	G	T	T	-	G	T	T	6
H5	-	-	-	G	T	T	-	C	T	G	1
H6	-	-	-	G	G	T	-	G	T	T	3
H7	-	-	-	G	T	T	-	G	T	T	6
H8	-	-	-	G	T	T	-	G	T	G	1
H9	T	-	-	G	T	T	-	G	T	T	5

Supplementary Table S20. Statistics of chloroplast haplotypes and diversity index of *C. tientaiensis* by cpDNA.

Population	Haplotype diversity (H_d)	Nucleotide diversity ($\pi \times 10^{-3}$)	Number of haplotypes	Composition and number of haplotypes
DPS	0.000±0.000	0.00±0.00	1	H9(5)
TTS	0.757±0.063	1.03±0.14	5	H4(6), H5(1), H6(3), H7(6), H8(1)
YTH	0.000±0.000	0.00±0.00	1	H1(2)
SST	0.140±0.087	0.09±0.06	3	H1(26), H2(1), H3(1)

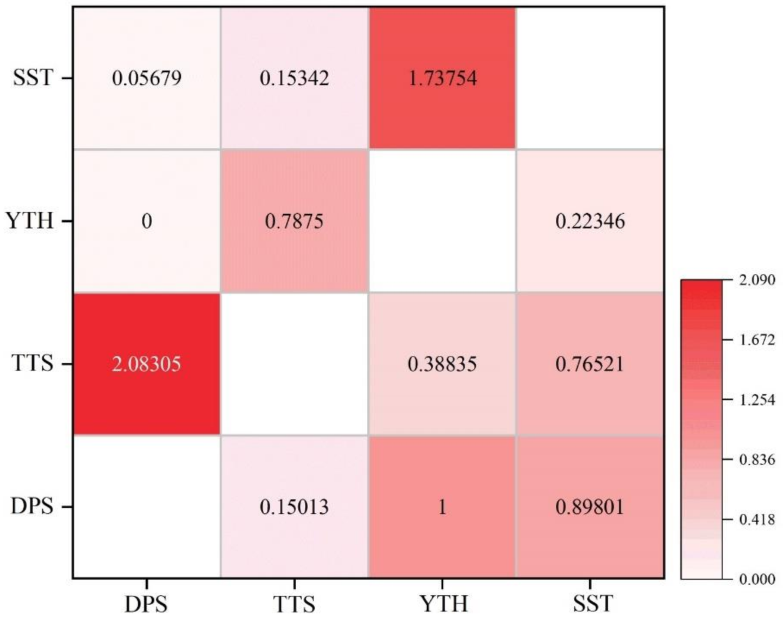

Supplementary Figure S1. *GammaSt* and *Nm* of *C. tientaiensis* based on cpDNA. Above the diagonal is *Nm*, and below the diagonal is *GammaSt.*

Supplementary Table S21. Neutral test results of *C. tientaiensis.*

	**Total population**	**Northern geographical group**	**Southern geographical group**
Tajima's *D*	-0.55923 (p>0.1)	-0.15861 (p>0.1)	-0.55923 (p>0.1)
Fu and Li's *D**	-1.06300 (p>0.1)	-1.42800 (p>0.1)	-1.06300 (p>0.1)
