# Supplementary material for: Regional environmental differences significantly affect the genetic structure and genetic differentiation of Carpinus tientaiensis Cheng, an endemic and extremely endangered species from China
Source: Front Plant Sci. 2024 Feb 9;15:1277173. doi: 10.3389/fpls.2024.1277173 (PMC10885731; doi:10.3389/fpls.2024.1277173)
Supplement: Supplementary file 2 [file Table_1.docx]

Supplementary Material

**Regional environmental differences significantly affect the genetic structure and genetic differentiation of *Carpinus tientaiensis* Cheng, an endemic and extremely endangered species from China**

**Runan Zhao ^1,2^, Qianqian He ^3^, Xiaojie Chu ^4^, Anguo He ^5^, Yuanlan Zhang ^2,6*^, and**

**Zunling Zhu ^1,2,7,^****^*^**

^1^ College of Landscape Architecture, Nanjing Forestry University, Nanjing, China

^2^ Co-Innovation Center for Sustainable Forestry in Southern China, Nanjing Forestry University, Nanjing, China

^3^ Research Center for Urban and Rural Living Environment, Zhijiang College of Zhejiang University of Technology, Shaoxing, China

^4^ College of Life Sciences, Zhejiang Normal University, Jinhua, China

^5^ Administration of Zhejiang Dapanshan National Nature Reserve, Pan’an, China

^6^ College of Life Sciences, Nanjing Forestry University, Nanjing, China

^7^ Jinpu Research Institute, Nanjing Forestry University, Nanjing, China

*** Correspondence:**

zhuzunling@njfu.edu.cn (Zunling Zhu); nlzyl2020@163.com (Yuanlan Zhang)

**Supplementary Table S1.** The geographical location and number of individuals of *C. tientaiensis.*

| **Population** | **Geographical location** | **Longitude** | **Latitude** | **Altitude (m)** | **Sample sizes** |
| --- | --- | --- | --- | --- | --- |
| DPS | Dapanshan National Nature Reserve, Pan'an County, Jinhua City, China | 120.5218 | 28.9708 | 1138 | 5 |
| TTS | Tiantai Mountain, Tiantai County, Tianzhou City, China | 121.0917 | 29.2568 | 901 | 17 |
| YTH | Yangtianhe, Qingtian County, Lishui City, China | 119.9907 | 28.2084 | 1249 | 2 |
| SST | Shangshantou, Jingning She Autonomous County, Lishui City, China | 119.6320 | 27.7823 | 1506 | 29 |

**Supplementary Table S2.** Reaction system.

| **Reagent** | **Contrast** **group** | **Treatment** **group** |
| --- | --- | --- |
| Control template DNA | 4 μl | - |
| Template DNA | - | 4 μl |
| Adapter | 1 μl | 1 μl |
| *Eco*R I/*Mse* I | 2 μl | 2 μl |
| 10×Reaction buffer | 2.5 μl | 2.5 μl |
| 10mM ATP | 2.5 μl | 2.5 μl |
| T4 Ligase | 1 μl | 1 μl |
| AFLP-Water | 7 μl | 7 μl |
| Total | 20 μl | 20 μl |

**Supplementary Table S3.** Joint sequences.

| **Connector** | **Sequence 1 (5’-3’)** | **Sequence 2 (5’-3’)** |
| --- | --- | --- |
| *Eco*R I | CTCGTAGACTGCGTACC | AATTGGTACGCAGTCTAC |
| *Mse* I | GACGATGAGTCCTGAG | TACTCAGGACTCAT |

**Supplementary Table S4.** Pre-amplification reaction system.

| **Reagent** | **Volume** |
| --- | --- |
| Template DNA | 2 μl |
| Pre-ampmix | 1 μl |
| dNTPs | 0.5 μl |
| 10×PCR buffer | 2.5 μl |
| Taq DNA polymease | 0.5 μl |
| ddH_2_O | 18.5 μl |
| Total | 20 μl |

**Supplementary Table S5.** Joint sequences.

| **Connector** | **Sequence 1(5’-3’)** | **Sequence 2(5’-3’)** |
| --- | --- | --- |
| *Eco*R I | CTCGTAGACTGCGTACC | AATTGGTACGCAGTCTAC |
| *Mse* I | GACGATGAGTCCTGAG | TACTCAGGACTCAT |

**Supplementary Table S6.** AFLP primer sequences.

| ***Ec*oR I (5ng/μl)** | |  | ***Mse* I (30ng/μl)** | |
| --- | --- | --- | --- | --- |
| **Code** | **Primers sequence (5’-3’)** |  | **Code** | **Primers sequence (5’-3’)** |
| *E-AAC* | GACTGCGTACCAATTCAAC |  | *M-CAA* | GATGAGTCCTGAGTAACAA |
| *E-AAG* | GACTGCGTACCAATTCAAG |  | *M-CAC* | GATGAGTCCTGAGTAACAC |
| *E-ACA* | GACTGCGTACCAATTCACA |  | *M-CAG* | GATGAGTCCTGAGTAACAG |
| *E-ACT* | GACTGCGTACCAATTCACT |  | *M-CAT* | GATGAGTCCTGAGTAACAT |
| *E-ACC* | GACTGCGTACCAATTCACC |  | *M-CTA* | GATGAGTCCTGAGTAACTA |
| *E-ACG* | GACTGCGTACCAATTCACG |  | *M-CTC* | GATGAGTCCTGAGTAACTC |
| *E-AGC* | GACTGCGTACCAATTCAGC |  | *M-CTG* | GATGAGTCCTGAGTAACTG |
| *E-AGG* | GACTGCGTACCAATTCAGG |  | *M-CTT* | GATGAGTCCTGAGTAACTT |

**Supplementary Table S7.** Amplification reaction system.

| **Reagent** | **Volume** |
| --- | --- |
| Pre amplified samples after dilution | 2 μl |
| 10×PCR buffer | 2.5 μl |
| dNTPs | 0.5 μl |
| *Eco*R I primer | 1 μl |
| *Mse* I primer | 1 μl |
| Taq enzyme | 0.5 μl |
| ddH_2_O | 17.5 μl |
| Total | 25 μl |

**Supplementary Table S8.** Primer combinations for amplification.

| **Primer pairs** | ***Eco*R I primers (5’-3’)** | ***Ms*e I primers (5’-3’)** |
| --- | --- | --- |
| *E-ACA/M-CAG* | GACTGCGTACCAATTCACA | GATGAGTCCTGAGTAACAG |
| *E-ACC/M-CTC* | GACTGCGTACCAATTCACC | GATGAGTCCTGAGTAACTC |
| *E-ACG/M-CAA* | GACTGCGTACCAATTCACG | GATGAGTCCTGAGTAACAA |
| *E-ACG/M-CAT* | GACTGCGTACCAATTCACG | GATGAGTCCTGAGTAACAT |
| *E-AGC/M-CAC* | GACTGCGTACCAATTCAGC | GATGAGTCCTGAGTAACAC |
| *E-AGC/M-CAG* | GACTGCGTACCAATTCAGC | GATGAGTCCTGAGTAACAG |
| *E-AGC/M-CTC* | GACTGCGTACCAATTCAGC | GATGAGTCCTGAGTAACTC |
| *E-AGC/M-CTG* | GACTGCGTACCAATTCAGC | GATGAGTCCTGAGTAACTG |

**Supplementary Table S9.** Primers and annealing temperature of cpDNA sequences.

| **Primers** | **Primer sequences (5’-3’)** | ***T*_A_ (℃)** | **Length (bp)** | **References** |
| --- | --- | --- | --- | --- |
| *trn*L-*trn*F | F: AAAATCGTGAGGGTTCAAGTC | 56 | 452 | Sang *et al*., 1997 |
|  | R: GATTTGAACTGGTGACACGAG |  |  |  |
| *trn*G | F: GGTAAAAGTGTGATTCGTTC | 58 | 603 | Nishizawa *et al*., 2000 |
|  | R: GTTTCATTCGGCTCCTTTAT |  |  |  |
| *rps*16 | F: GTGGTAGAAAGCAACGTGCGACTT | 56 | 803 | Oxelman *et al*., 1997 |
|  | R: GGTTTAGACATTACTTCGTTGA |  |  |  |
| *atp*B-*rbc*L | F: TAGTTTCTGTTTGTGGTGACAT | 55 | 718 | Okaura *et al*., 2007 |
|  | R: AAGTAGTAGGATTGGTTCTCAT |  |  |  |
| *psb*A-*trn*H | F: ACGGGAATTGAACCCGCGCA | 57 | 514 | Okaura *et al*., 2007 |
|  | R: TATTATTAACCGTGCTAACC |  |  |  |
| *pet*B*-pet*D | F: CAATCCACTTTGACTCGTTTT | 60 | 612 | Dong *et al*., 2012 |
|  | R: GGTTCACCAATCATTGATGGTTC |  |  |  |
| *trn*S(GCU)-*trn*G(UCC) | F: GCCGCTTTAGTCCACTCAGC | 60 | 607 | Hamilton, 1999 |
|  | R: GAACGAATCACACTTTTACCAC |  |  |  |
| *trn*S(GCU)-*trn*T(GGU) | F: GAGATGGCCGAGTGGTTGAA | 60 | 902 | Kanno *et al*., 2004 |
|  | R: CCCGCTTAGCTCAGAGGTTAGAG |  |  |  |

**References**

Dong WP, Liu J, Yu J, *et al*., 2012. Highly Variable Chloroplast Markers for Evaluating Plant Phylogeny at Low Taxonomic Levels and for DNA Barcoding. Plos one. 7: e35071.

Hamilton MB, 1999. Four primer pairs for the amplification of chloroplast intergenic regions with intraspecific variation. Mol. Ecol. 8(3): 521-523. In: 1999. Primer Notes. Mol. Ecol. 8: 513-525.

Kanno M, Yokoyama J, Suyama Y, *et al*., 2004. Geographical distribution of two haplotypes of chloroplast DNA in four oak species (*Quercus*) in Japan. J. Plant Res. 117: 311-317.

Nishizawa T, Watano Y, 2000. Primer pairs suitable for PCR-SSCP analysis of chloroplast DNA in angiosperms. J. Phytogeogr. Taxon. 48: 63-66.

Okaura T, Quang ND, Ubukata M, *et al*., 2007. Phylogeographic structure and late Quaternary population history of the Japanese oak *Quercus mongolica* var. *crispula* and related species revealed by chloroplast DNA variation. Genes Genet. Syst. 82: 465-477.

Oxelman B, Lidén M, Berglund D, 1997. Chloroplast rps16 intron phylogeny of the tribe Sileneae (Caryophyllaceae). Plant Syst. Evol. 206: 393-410.

Sang T, Crawford DJ, Stuessy TF, 1997. Chloroplast DNA phylogeny, reticulate evolution and biogeography of *Paeonia* (Paeoniaceae). Am. J. Bot. 84: 1120-1136.

**Supplementary Table S10.** The 19 bioclimatic variables and jackknife analysis results of MaxEnt model.

| **Variable** | **Variable description** | **Unit** | **Contribution Rate (%)** |
| --- | --- | --- | --- |
| bio11 | Mean temperature of coldest quarter | ℃ | 28.1 |
| bio10 | Mean temperature of warmest quarter | ℃ | 21.4 |
| bio17 | Precipitation of the driest quarter | mm | 21.1 |
| bio03 | Isothermality (bio2 /bio7 × 100) | % | 12.6 |
| bio08 | Mean temperature of wettest quarter | ℃ | 6.7 |
| bio15 | Coefficient of variation (CV) of precipitation seasonality | % | 3.8 |
| bio16 | Precipitation of wettest quarter | mm | 2.2 |
| bio05 | Max temperature of the warmest month | ℃ | 1.0 |
| bio19 | Precipitation of the coldest quarter | mm | 0.7 |
| bio12 | Annual precipitation | mm | 0.7 |
| bio09 | Mean temperature of driest quarter | ℃ | 0.6 |
| bio06 | Min temperature of coldest month | ℃ | 0.5 |
| bio01 | Annual mean temperature | ℃ | 0.3 |
| bio18 | Precipitation of the coldest quarter | mm | 0.1 |
| bio04 | Standard deviation (SD) of temperature seasonality | % | 0.1 |
| bio14 | Precipitation of the driest month | mm | 0 |
| bio13 | Precipitation of wettest month | mm | 0 |
| bio02 | Mean diurnal temperature range | ℃ | 0 |
| bio07 | Range of annual temperature | ℃ | 0 |

**Supplementary Table S11.** The 12 effective distribution data used in the MaxEnt model.

| **Species** | **Longitude** | **Latitude** | **Location** |
| --- | --- | --- | --- |
| *C. tientaiensis* | 121.09915 | 29.25046 | Tianzhou City, Zhejiang Province, China |
| *C. tientaiensis* | 121.09314 | 29.25175 | Tianzhou City, Zhejiang Province, China |
| *C. tientaiensis* | 121.08853 | 29.25437 | Tianzhou City, Zhejiang Province, China |
| *C. tientaiensis* | 121.09706 | 29.25392 | Tianzhou City, Zhejiang Province, China |
| *C. tientaiensis* | 121.09170 | 29.25683 | Tianzhou City, Zhejiang Province, China |
| *C. tientaiensis* | 120.52585 | 28.96782 | Jinhua City, Zhejiang Province, China |
| *C. tientaiensis* | 120.44910 | 28.84516 | Jinhua City, Zhejiang Province, China |
| *C. tientaiensis* | 120.52304 | 28.97513 | Jinhua City, Zhejiang Province, China |
| *C. tientaiensis* | 120.54056 | 28.90278 | Jinhua City, Zhejiang Province, China |
| *C. tientaiensis* | 119.99075 | 28.20843 | Lishui City, Zhejiang Province, China |
| *C. tientaiensis* | 119.63037 | 27.78506 | Lishui City, Zhejiang Province, China |
| *C. tientaiensis* | 119.62528 | 27.78528 | Lishui City, Zhejiang Province, China |

**Supplementary Table S12.** The correlation coefficient between the 19 bioclimatic variables.

|  | **bio01** | **bio02** | **bio03** | **bio04** | **bio05** | **bio06** | **bio07** | **bio08** | **bio09** | **bio10** | **bio11** | **bio12** | **bio13** | **bio14** | **bio15** | **bio16** | **bio17** | **bio18** | **bio19** |
| --- | --- | --- | --- | --- | --- | --- | --- | --- | --- | --- | --- | --- | --- | --- | --- | --- | --- | --- | --- |
| **bio01** | 1 | -0.252 | 0.543 | -0.605 | 0.605 | 0.919 | -0.600 | 0.586 | 0.922 | 0.761 | 0.947 | 0.448 | 0.482 | 0.172 | 0.296 | 0.536 | 0.165 | 0.500 | 0.255 |
| **bio02** | -0.252 | 1 | 0.080 | 0.486 | 0.197 | -0.497 | 0.610 | -0.031 | -0.359 | 0.062 | -0.384 | -0.371 | -0.301 | -0.188 | -0.135 | -0.358 | -0.117 | -0.428 | -0.095 |
| **bio03** | 0.543 | 0.080 | 1 | -0.814 | -0.145 | 0.648 | -0.734 | 0.253 | 0.620 | 0.009 | 0.703 | 0.443 | 0.560 | -0.114 | 0.577 | 0.590 | -0.126 | 0.654 | -0.002 |
| **bio04** | -0.605 | 0.486 | -0.814 | 1 | 0.237 | -0.846 | 0.983 | -0.166 | -0.748 | 0.054 | -0.828 | -0.610 | -0.640 | -0.030 | -0.550 | -0.709 | 0.026 | -0.767 | -0.086 |
| **bio05** | 0.605 | 0.197 | -0.145 | 0.237 | 1 | 0.288 | 0.253 | 0.453 | 0.385 | 0.956 | 0.332 | -0.039 | -0.056 | 0.194 | -0.208 | -0.044 | 0.241 | -0.195 | 0.253 |
| **bio06** | 0.919 | -0.497 | 0.648 | -0.846 | 0.288 | 1 | -0.854 | 0.449 | 0.936 | 0.469 | 0.990 | 0.563 | 0.587 | 0.139 | 0.430 | 0.659 | 0.101 | 0.665 | 0.198 |
| **bio07** | -0.600 | 0.610 | -0.734 | 0.983 | 0.253 | -0.854 | 1 | -0.207 | -0.736 | 0.046 | -0.819 | -0.590 | -0.624 | -0.035 | -0.547 | -0.689 | 0.029 | -0.778 | -0.062 |
| **bio08** | 0.586 | -0.031 | 0.253 | -0.166 | 0.453 | 0.449 | -0.207 | 1 | 0.388 | 0.598 | 0.473 | -0.157 | -0.009 | -0.326 | 0.416 | -0.003 | -0.348 | 0.237 | -0.337 |
| **bio09** | 0.922 | -0.359 | 0.620 | -0.748 | 0.385 | 0.936 | -0.736 | 0.388 | 1 | 0.548 | 0.953 | 0.602 | 0.605 | 0.270 | 0.285 | 0.660 | 0.250 | 0.587 | 0.381 |
| **bio10** | 0.761 | 0.062 | 0.009 | 0.054 | 0.956 | 0.469 | 0.046 | 0.598 | 0.548 | 1 | 0.515 | 0.065 | 0.078 | 0.189 | -0.079 | 0.092 | 0.225 | -0.001 | 0.244 |
| **bio11** | 0.947 | -0.384 | 0.703 | -0.828 | 0.332 | 0.990 | -0.819 | 0.473 | 0.953 | 0.515 | 1 | 0.565 | 0.597 | 0.142 | 0.419 | 0.662 | 0.114 | 0.657 | 0.222 |
| **bio12** | 0.448 | -0.371 | 0.443 | -0.610 | -0.039 | 0.563 | -0.590 | -0.157 | 0.602 | 0.065 | 0.565 | 1 | 0.899 | 0.629 | 0.002 | 0.922 | 0.625 | 0.765 | 0.690 |
| **bio13** | 0.482 | -0.301 | 0.560 | -0.640 | -0.056 | 0.587 | -0.624 | -0.009 | 0.605 | 0.078 | 0.597 | 0.899 | 1 | 0.375 | 0.334 | 0.972 | 0.376 | 0.873 | 0.461 |
| **bio14** | 0.172 | -0.188 | -0.114 | -0.030 | 0.194 | 0.139 | -0.035 | -0.326 | 0.270 | 0.189 | 0.142 | 0.629 | 0.375 | 1 | -0.618 | 0.389 | 0.976 | 0.099 | 0.941 |
| **bio15** | 0.296 | -0.135 | 0.577 | -0.550 | -0.208 | 0.430 | -0.547 | 0.416 | 0.285 | -0.079 | 0.419 | 0.002 | 0.334 | -0.618 | 1 | 0.337 | -0.648 | 0.534 | -0.565 |
| **bio16** | 0.536 | -0.358 | 0.590 | -0.709 | -0.044 | 0.659 | -0.689 | -0.003 | 0.660 | 0.092 | 0.662 | 0.922 | 0.972 | 0.389 | 0.337 | 1 | 0.386 | 0.889 | 0.468 |
| **bio17** | 0.165 | -0.117 | -0.126 | 0.026 | 0.241 | 0.101 | 0.029 | -0.348 | 0.250 | 0.225 | 0.114 | 0.625 | 0.376 | 0.976 | -0.648 | 0.386 | 1 | 0.079 | 0.957 |
| **bio18** | 0.500 | -0.428 | 0.654 | -0.767 | -0.195 | 0.665 | -0.778 | 0.237 | 0.587 | -0.001 | 0.657 | 0.765 | 0.873 | 0.099 | 0.534 | 0.889 | 0.079 | 1 | 0.134 |
| **bio19** | 0.255 | -0.095 | -0.002 | -0.086 | 0.253 | 0.198 | -0.062 | -0.337 | 0.381 | 0.244 | 0.222 | 0.690 | 0.461 | 0.941 | -0.565 | 0.468 | 0.957 | 0.134 | 1 |

**Supplementary Table S13.** Contribution rate of the 9 bioclimatic variables in the MaxEnt model in current period.

| **Variable** | **Variable description** | **Unit** | **Contribution Rate (%)** |
| --- | --- | --- | --- |
| bio11 | Mean temperature of coldest quarter | ℃ | 25.2 |
| bio10 | Mean temperature of warmest quarter | ℃ | 23.5 |
| bio17 | Precipitation of the driest quarter | mm | 20.0 |
| bio03 | Isothermality (bio2 /bio7 × 100) | % | 13.9 |
| bio08 | Mean temperature of wettest quarter | ℃ | 11.0 |
| bio15 | CV of precipitation seasonality | % | 2.7 |
| bio16 | Precipitation of wettest quarter | mm | 2.1 |
| bio04 | SD of temperature seasonality | % | 1.4 |
| bio02 | Mean diurnal temperature range | ℃ | 0.1 |

**Supplementary Table S14.** The area under the curve (AUC), minimum training presence area, maximum test sensitivity plus specificity area, and equate entropy of thresholded and original distributions area of MaxEnt models under different regularization multipliers.

| **Regularization multipliers** | **Training AUC** | **Test AUC** | **Minimum training presence area** | **Maximum test sensitivity plus specificity area** | **Equate entropy of thresholded and original distributions area** |
| --- | --- | --- | --- | --- | --- |
| 0.25 | 0.9994 | 0.9993 | 0.0014 | 0.0007 | 0.0026 |
| 0.3 | 0.9994 | 0.9993 | 0.0014 | 0.0007 | 0.0028 |
| 0.4 | 0.9994 | 0.9993 | 0.0015 | 0.0008 | 0.0033 |
| 0.5 | 0.9994 | 0.9993 | 0.0015 | 0.0008 | 0.0037 |
| 0.6 | 0.9993 | 0.9992 | 0.0015 | 0.0008 | 0.0042 |
| 0.7 | 0.9993 | 0.9992 | 0.0016 | 0.0009 | 0.0046 |
| 0.75 | 0.9993 | 0.9992 | 0.0016 | 0.0009 | 0.0049 |
| 1.0 | 0.9993 | 0.9991 | 0.0018 | 0.001 | 0.0062 |
| 1.25 | 0.9991 | 0.9991 | 0.0022 | 0.0011 | 0.0076 |
| 1.5 | 0.9991 | 0.999 | 0.0024 | 0.0011 | 0.0091 |
| 1.75 | 0.999 | 0.9989 | 0.0028 | 0.0013 | 0.0106 |
| 2.0 | 0.9988 | 0.9988 | 0.0035 | 0.0015 | 0.0122 |

**Supplementary Table S15.** The number of loci amplified by each primer pair.

| **Primer pairs** | **DPS** | | **TTS** | | **YTH** | | **SST** | | **Total** | | **Loci** |
| --- | --- | --- | --- | --- | --- | --- | --- | --- | --- | --- | --- |
|  | ***N*** | ***PPBs*** | ***N*** | ***PPBs*** | ***N*** | ***PPBs*** | ***N*** | ***PPBs*** | ***N*** | ***PPBs*** |  |
| *E-ACA/M-CAG* | 80 | 37.04% | 122 | 56.48% | 40 | 18.52% | 129 | 59.72% | 162 | 75% | 216 |
| *E-ACC/M-CTC* | 81 | 37.50% | 141 | 65.28% | 32 | 14.81% | 150 | 69.44% | 178 | 82.41% | 216 |
| *E-ACG/M-CAA* | 119 | 55.09% | 141 | 65.28% | 44 | 20.37% | 159 | 73.61% | 178 | 82.41% | 216 |
| *E-ACG/M-CAT* | 63 | 29.17% | 104 | 48.15% | 29 | 13.43% | 129 | 59.72% | 153 | 70.83% | 216 |
| *E-AGC/M-CAC* | 81 | 37.50% | 115 | 53.24% | 24 | 11.11% | 143 | 66.20% | 156 | 72.22% | 216 |
| *E-AGC/M-CAG* | 72 | 33.33% | 111 | 51.39% | 23 | 10.65% | 118 | 54.63% | 155 | 71.76% | 216 |
| *E-AGC/M-CTC* | 105 | 48.61% | 144 | 66.67% | 61 | 28.24% | 139 | 64.35% | 179 | 82.87% | 216 |
| *E-AGC/M-CTG* | 68 | 31.48% | 108 | 50.00% | 25 | 11.57% | 130 | 60.19% | 155 | 71.76% | 216 |
| Mean | 84 | 38.72% | 123 | 57.06% | 35 | 16.09% | 137 | 63.48% | 165 | 76.16% | 216 |
| Total | 669 | 38.72% | 986 | 57.06% | 278 | 16.09% | 1097 | 63.48% | 1316 | 76.16% | 1728 |

**Supplementary Table S16.** Genetic diversity of *C. tientaiensis.*

| **Primer pairs** | **Population** | ***Na*** | ***Ne*** | ***H*** | ***I*** |
| --- | --- | --- | --- | --- | --- |
| *E-ACA/M-CAG* | DPS | 1.3704±0.4840 | 1.2008±0.3185 | 0.1214±0.1759 | 0.1854±0.2578 |
|  | TTS | 1.5648±0.4969 | 1.2317±0.3134 | 0.1450±0.1728 | 0.2290±0.2502 |
|  | YTH | 1.1852±0.3894 | 1.1309±0.2753 | 0.0767±0.1613 | 0.1120±0.2354 |
|  | SST | 1.5972±0.4916 | 1.2249±0.3124 | 0.1403±0.1727 | 0.2224±0.2485 |
|  | Total | 1.7500±0.4340 | 1.2449±0.3140 | 0.1548±0.1695 | 0.2495±0.2400 |
| *E-ACC/M-CTC* | DPS | 1.3750±0.4852 | 1.2103±0.3291 | 0.1257±0.1801 | 0.1908±0.2626 |
|  | TTS | 1.6528±0.4772 | 1.2449±0.3204 | 0.1533±0.1722 | 0.2450±0.2446 |
|  | YTH | 1.1481±0.3561 | 1.1048±0.2518 | 0.0614±0.1475 | 0.0896±0.2153 |
|  | SST | 1.6944±0.4617 | 1.2384±0.3098 | 0.1512±0.1684 | 0.2436±0.2399 |
|  | Total | 1.8241±0.3816 | 1.2624±0.3146 | 0.1668±0.1677 | 0.2708±0.2334 |
| *E-ACG/M-CAA* | DPS | 1.5509±0.4986 | 1.3114±0.3616 | 0.1853±0.1930 | 0.2809±0.2772 |
|  | TTS | 1.6528±0.4772 | 1.2794±0.3380 | 0.1717±0.1800 | 0.2696±0.2550 |
|  | YTH | 1.2037±0.4037 | 1.1440±0.2854 | 0.0844±0.1672 | 0.1232±0.2441 |
|  | SST | 1.7361±0.4418 | 1.2681±0.3139 | 0.1709±0.1672 | 0.2751±0.2363 |
|  | Total | 1.8241±0.3816 | 1.2909±0.3162 | 0.1853±0.1672 | 0.2980±0.2328 |
| *E-ACG/M-CAT* | DPS | 1.2917±0.4556 | 1.1349±0.2640 | 0.0850±0.1488 | 0.1335±0.2226 |
|  | TTS | 1.4815±0.5008 | 1.1879±0.2972 | 0.1180±0.1639 | 0.1879±0.2392 |
|  | YTH | 1.1343±0.3417 | 1.0949±0.2416 | 0.0556±0.1415 | 0.0812±0.2066 |
|  | SST | 1.5972±0.4916 | 1.1893±0.2920 | 0.1203±0.1611 | 0.1956±0.2321 |
|  | Total | 1.7083±0.4556 | 1.2075±0.2981 | 0.1325±0.1625 | 0.2165±0.2321 |
| *E-AGC/M-CAC* | DPS | 1.3750±0.4852 | 1.1803±0.2913 | 0.1131±0.1642 | 0.1763±0.2440 |
|  | TTS | 1.5324±0.5001 | 1.1832±0.2844 | 0.1176±0.1579 | 0.1910±0.2302 |
|  | YTH | 1.1111±0.3150 | 1.0786±0.2227 | 0.0460±0.1305 | 0.0672±0.1905 |
|  | SST | 1.6620±0.4741 | 1.2158±0.3022 | 0.1376±0.1640 | 0.2237±0.2343 |
|  | Total | 1.7222±0.4489 | 1.2154±0.2939 | 0.1399±0.1574 | 0.2316±0.2234 |

**Supplementary Table S16.** Cont.

| **Primer pairs** | **Population** | ***Na*** | ***Ne*** | ***H*** | ***I*** |
| --- | --- | --- | --- | --- | --- |
| *E-AGC/M-CAG* | DPS | 1.3333±0.4725 | 1.1708±0.2957 | 0.1049±0.1654 | 0.1617±0.2442 |
|  | TTS | 1.5139±0.5010 | 1.1869±0.2885 | 0.1195±0.1594 | 0.1929±0.2333 |
|  | YTH | 1.1065±0.3092 | 1.0753±0.2186 | 0.0441±0.1281 | 0.0644±0.1870 |
|  | SST | 1.5463±0.4990 | 1.1751±0.2734 | 0.1140±0.1536 | 0.1865±0.2257 |
|  | Total | 1.7176±0.4512 | 1.1915±0.2714 | 0.1268±0.1521 | 0.2111±0.2199 |
| *E-AGC/M-CTC* | DPS | 1.4861±0.5010 | 1.2573±0.3337 | 0.1569±0.1834 | 0.2406±0.2674 |
|  | TTS | 1.6667±0.4725 | 1.2530±0.3014 | 0.1627±0.1667 | 0.2607±0.2401 |
|  | YTH | 1.2824±0.4512 | 1.1997±0.3191 | 0.1170±0.1869 | 0.1708±0.2729 |
|  | SST | 1.6435±0.4801 | 1.2083±0.2927 | 0.1341±0.1610 | 0.2186±0.2322 |
|  | Total | 1.8287±0.3776 | 1.2503±0.2987 | 0.1625±0.1613 | 0.2661±0.2268 |
| *E-AGC/M-CTG* | DPS | 1.3148±0.4655 | 1.1650±0.2937 | 0.1009±0.1646 | 0.1549±0.2429 |
|  | TTS | 1.5000±0.5012 | 1.1776±0.2827 | 0.1136±0.1580 | 0.1835±0.2311 |
|  | YTH | 1.1157±0.3207 | 1.0818±0.2267 | 0.0479±0.1328 | 0.0700±0.1939 |
|  | SST | 1.6019±0.4907 | 1.2016±0.2877 | 0.1299±0.1614 | 0.2103±0.2349 |
|  | Total | 1.7176±0.4512 | 1.2016±0.2782 | 0.1324±0.1559 | 0.2186±0.2249 |
| Mean | DPS | 1.3872±0.0825 | 1.2039±0.0529 | 0.1242±0.0304 | 0.1905±0.0451 |
|  | TTS | 1.5705±0.0711 | 1.2181±0.0364 | 0.1377±0.0217 | 0.2200±0.033 |
|  | YTH | 1.1609±0.0565 | 1.1138±0.0399 | 0.0666±0.0234 | 0.0973±0.0342 |
|  | SST | 1.6348±0.0574 | 1.2152±0.0273 | 0.1373±0.0167 | 0.2220±0.0260 |
|  | Total | 1.7616±0.0509 | 1.2331±0.0323 | 0.1501±0.0192 | 0.2453±0.0291 |

**Supplementary Table S17.** Genetic diversity, genetic differentiation coefficient, and gene flow of *C. tientaiensis.*

| **Primer pairs** | ***Ht*** | ***Hs*** | ***Gst*** | ***Nm*** |
| --- | --- | --- | --- | --- |
| *E-ACA/ M-CAG* | 0.1513±0.0269 | 0.1209±0.0163 | 0.2012 | 1.9849 |
| *E-ACC/ M-CTC* | 0.1647±0.0292 | 0.1229±0.0151 | 0.2538 | 1.4697 |
| *E-ACG/M-CAA* | 0.1944±0.0313 | 0.1531±0.0184 | 0.2126 | 1.8515 |
| *E-ACG/M-CAT* | 0.1262±0.0261 | 0.0947±0.0144 | 0.2491 | 1.5070 |
| *E-AGC/M-CAC* | 0.1384±0.0262 | 0.1036±0.0132 | 0.2519 | 1.4848 |
| *E-AGC/M-CAG* | 0.1273±0.0254 | 0.0956±0.0129 | 0.2489 | 1.5087 |
| *E-AGC/M-CTC* | 0.1847±0.0294 | 0.1427±0.0175 | 0.2274 | 1.6988 |
| *E-AGC/M-CTG* | 0.1260±0.0249 | 0.0981±0.0138 | 0.2217 | 1.7550 |
| Mean | 0.1516±0.0254 | 0.1165±0.0209 | 0.2333 | 1.6576 |

**Supplementary Table S18.** Analysis of molecular variance (AMOVA).

| **Source of variation** | | **Quadratic sum** | **Variance** | **Percentage (%)** | **Fixed coefficient** |
| --- | --- | --- | --- | --- | --- |
| AFLP | Among populations | 2098.459 | 26.6809 | 15.47503 | 0.15475  (*P* ＜ 0.00001) |
|  | Within populations | 14864.635 | 145.73172 | 84.52497 |  |
|  | Total | 16963.094 | 172.41262 |  |  |
| cpDNA | Among populations | 57.457 | 1.83486 | 85.55536 | 0.85555  (*P* ＜ 0.00001) |
|  | Within populations | 14.870 | 0.30979 | 14.44464 |  |
|  | Total | 72.327 | 2.14465 |  |  |

**Supplementary Table S19.** Nine haplotypes identified based on 2 singleton variable sites and 8 parsimony information sites.

| **Haplotypes** | ***trn*L-*trn*F** | |  | ***trn*G** | | |  | ***psb*A-*trn*H** | | | | | **Number** |
| --- | --- | --- | --- | --- | --- | --- | --- | --- | --- | --- | --- | --- | --- |
|  | **348** | **349** |  | **605** | **902** | **1037** |  | **1279** | **1396** | **1534** | **1540** | **1533** |  |
| H1 | T | T |  | - | A | T |  | C | T | G | T | T | 28 |
| H2 | T | T |  | - | A | T |  | C | T | G | G | T | 1 |
| H3 | T | T |  | - | A | G |  | C | T | G | T | T | 1 |
| H4 | T | - |  | A | G | T |  | T | - | G | T | T | 6 |
| H5 | - | - |  | - | G | T |  | T | - | C | T | G | 1 |
| H6 | - | - |  | - | G | G |  | T | - | G | T | T | 3 |
| H7 | - | - |  | - | G | T |  | T | - | G | T | T | 6 |
| H8 | - | - |  | - | G | T |  | T | - | G | T | G | 1 |
| H9 | T | - |  | - | G | T |  | T | - | G | T | T | 5 |

**Supplementary Table S20.** Statistics of chloroplast haplotypes and diversity index of *C. tientaiensis* by cpDNA.

| **Population** | **Haplotype diversity (*H*_d_)** | **Nucleotide diversity (π × 10^-3^)** | **Number of haplotypes** | **Composition and number of haplotypes** |
| --- | --- | --- | --- | --- |
| DPS | 0.000±0.000 | 0.00±0.00 | 1 | H9(5) |
| TTS | 0.757±0.063 | 1.03±0.14 | 5 | H4(6), H5(1), H6(3), H7(6), H8(1) |
| YTH | 0.000±0.000 | 0.00±0.00 | 1 | H1(2) |
| SST | 0.140±0.087 | 0.09±0.06 | 3 | H1(26), H2(1), H3(1) |


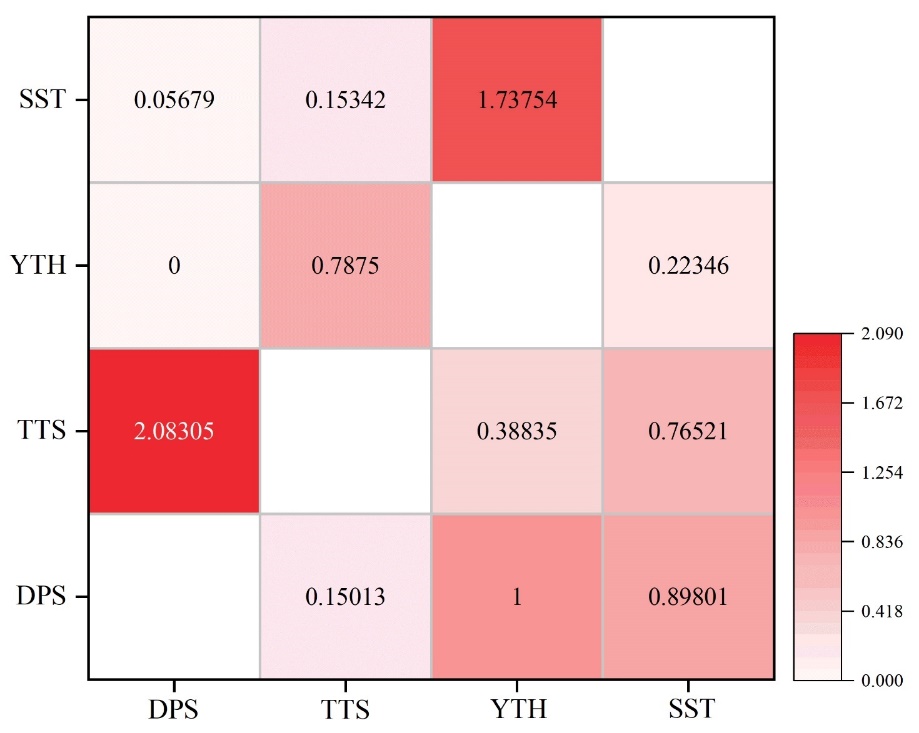


**Supplementary Figure S1.** *GammaSt* and *Nm* of *C. tientaiensis* based on cpDNA. Above the diagonal is *Nm*, and below the diagonal is *GammaSt.*

**Supplementary Table S21.** Neutral test results of *C. tientaiensis.*

|  | **Total population** | **Northern geographical group** | **Southern geographical group** |
| --- | --- | --- | --- |
| Tajima’s *D* | -0.55923 (*p*＞0.1) | -0.15861 (*p*＞0.1) | -0.55923 (*p*＞0.1) |
| Fu and Li's *D** | -1.06300 (*p*＞0.1) | -1.42800 (*p*＞0.1) | -1.06300 (*p*＞0.1) |
